# Supplementary figures and images for: Haplotypes spanning centromeric regions reveal persistence of large blocks of archaic DNA
Source: eLife. 2019 Jun 25;8:e42989. doi: 10.7554/eLife.42989 (PMC6592686; doi:10.7554/eLife.42989)

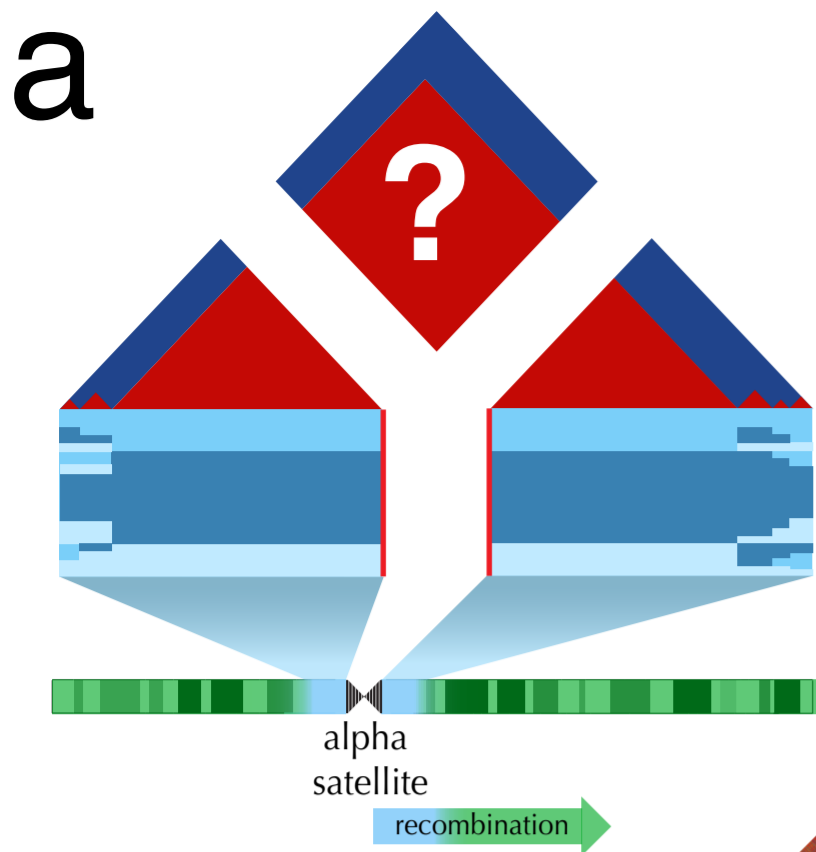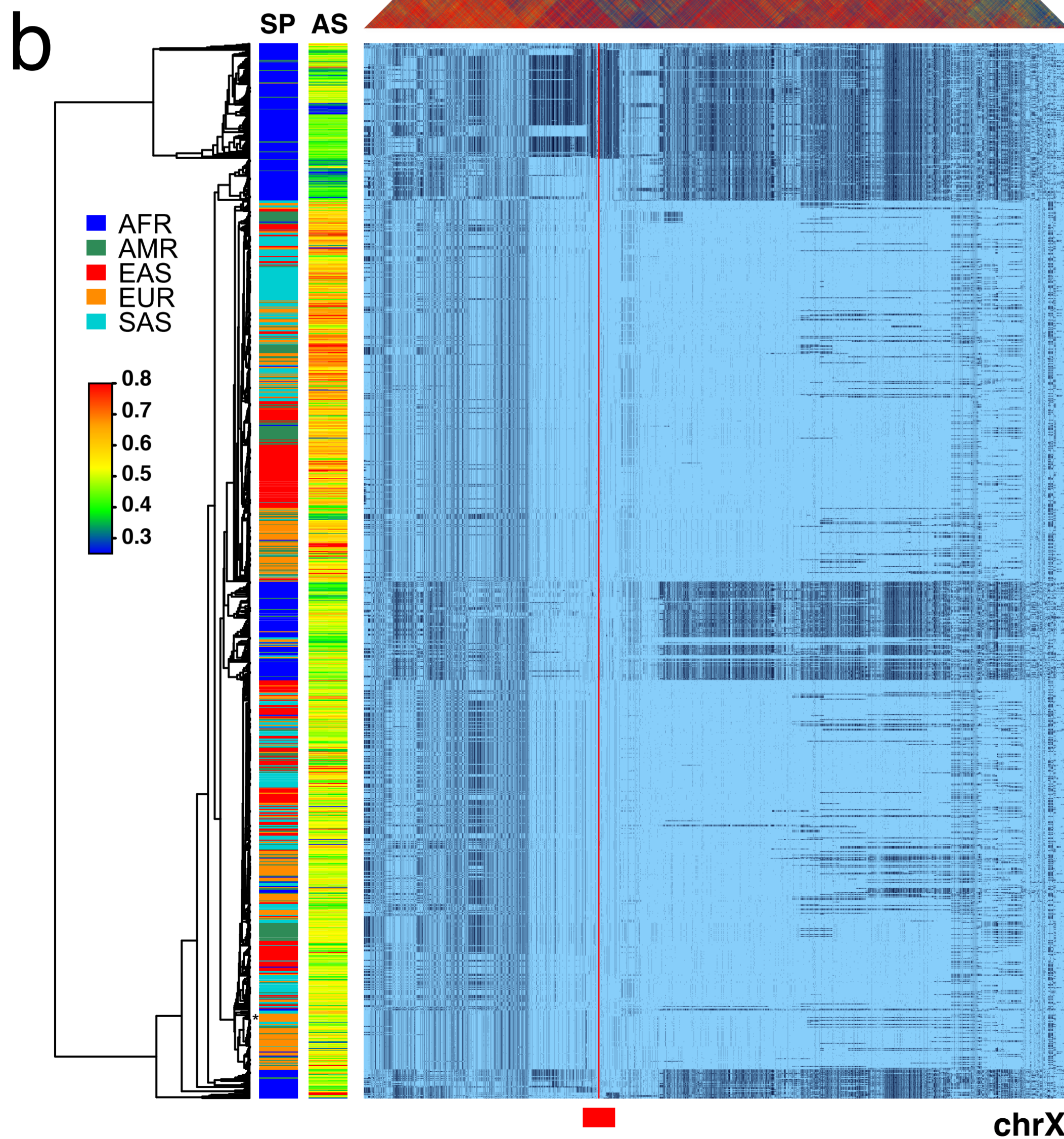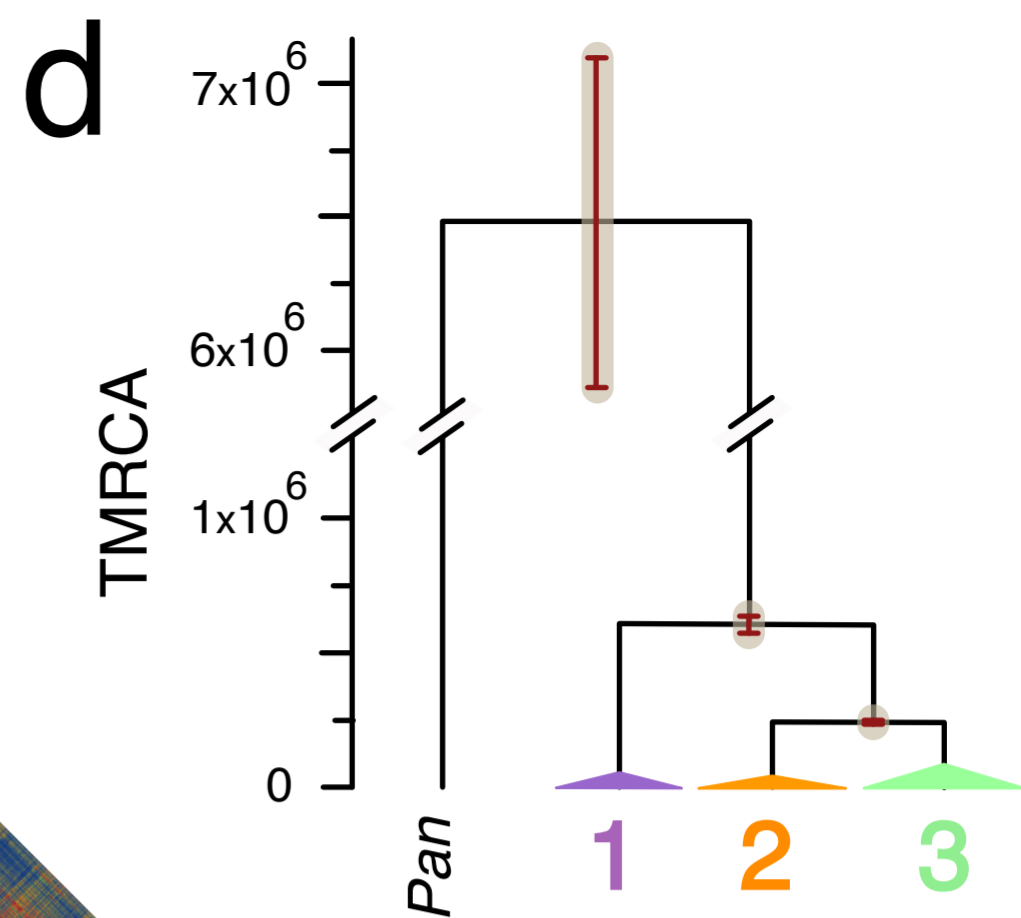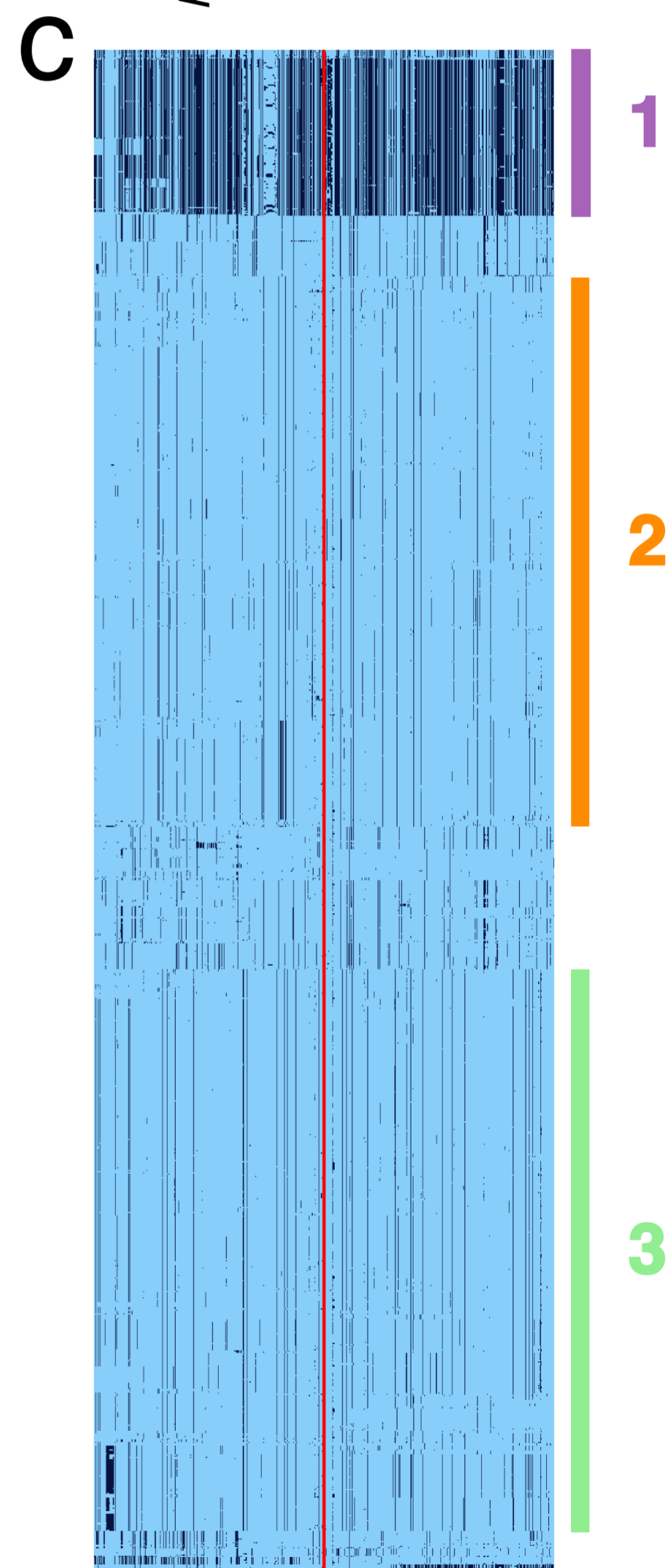

Supplement: Figure 1—source data 2. [file elife-42989-fig1-data2.pdf]

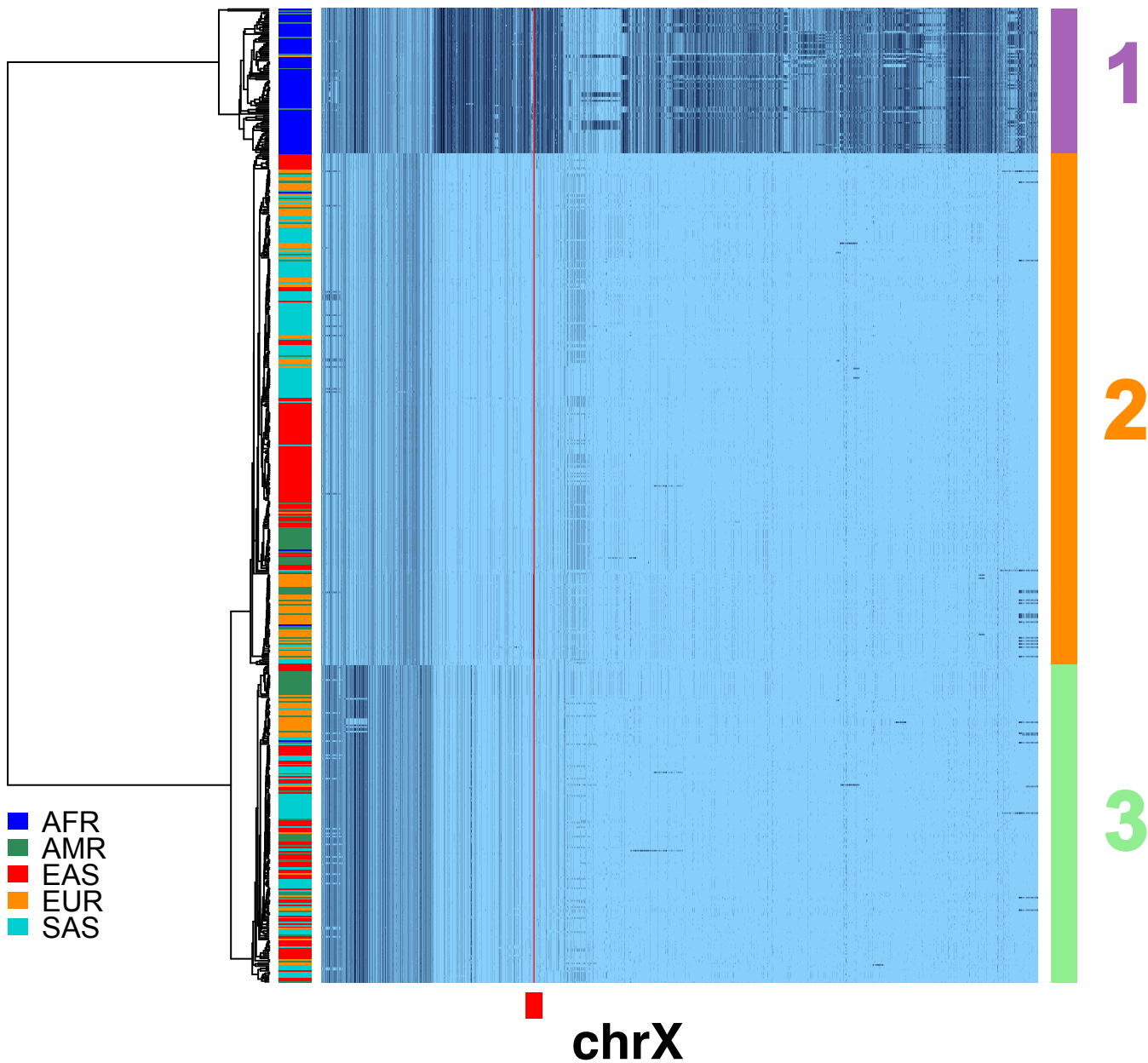

Supplement: Figure 1—figure supplement 2—source data 1. [file elife-42989-fig1-figsupp2-data1.pdf]

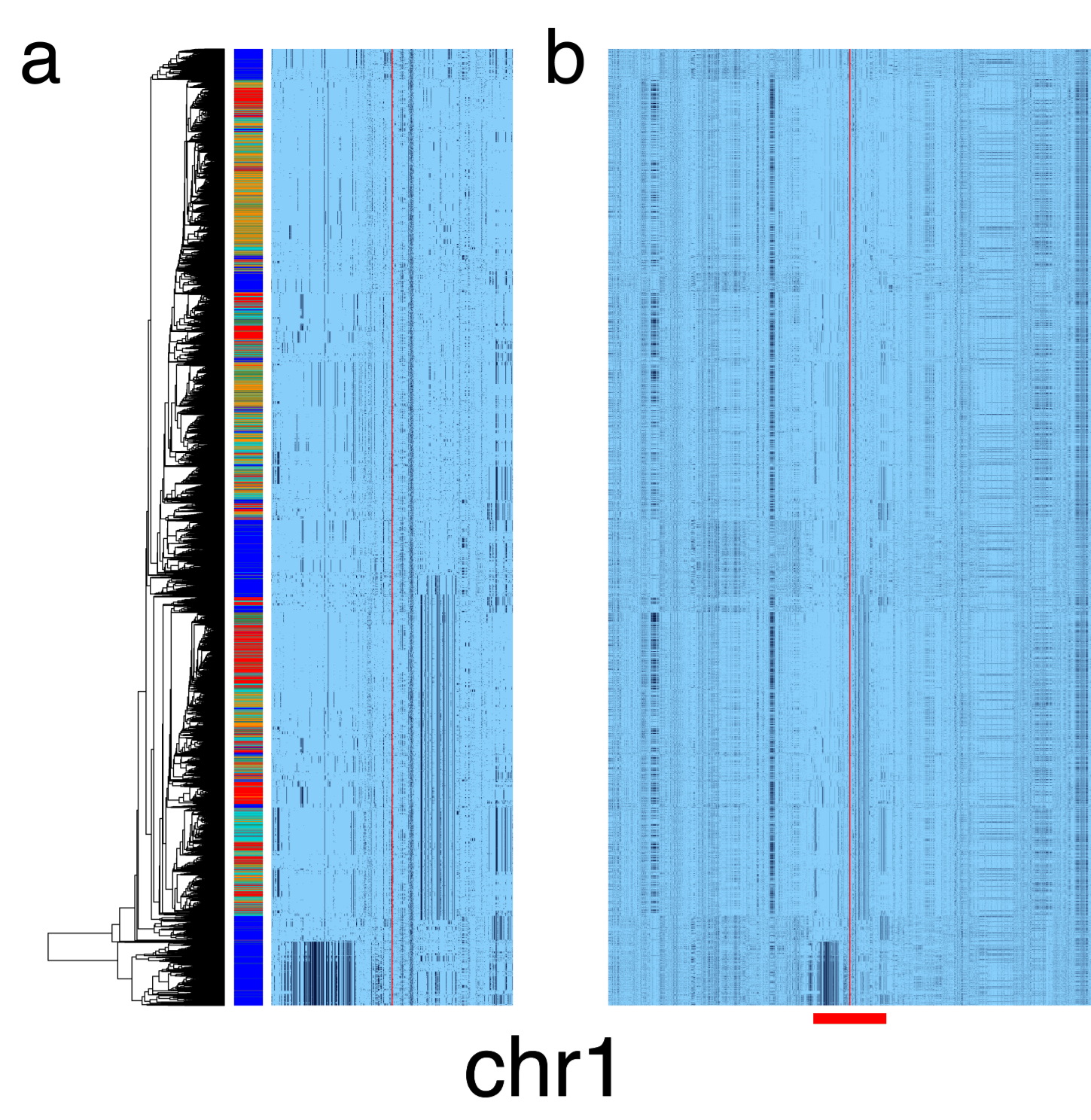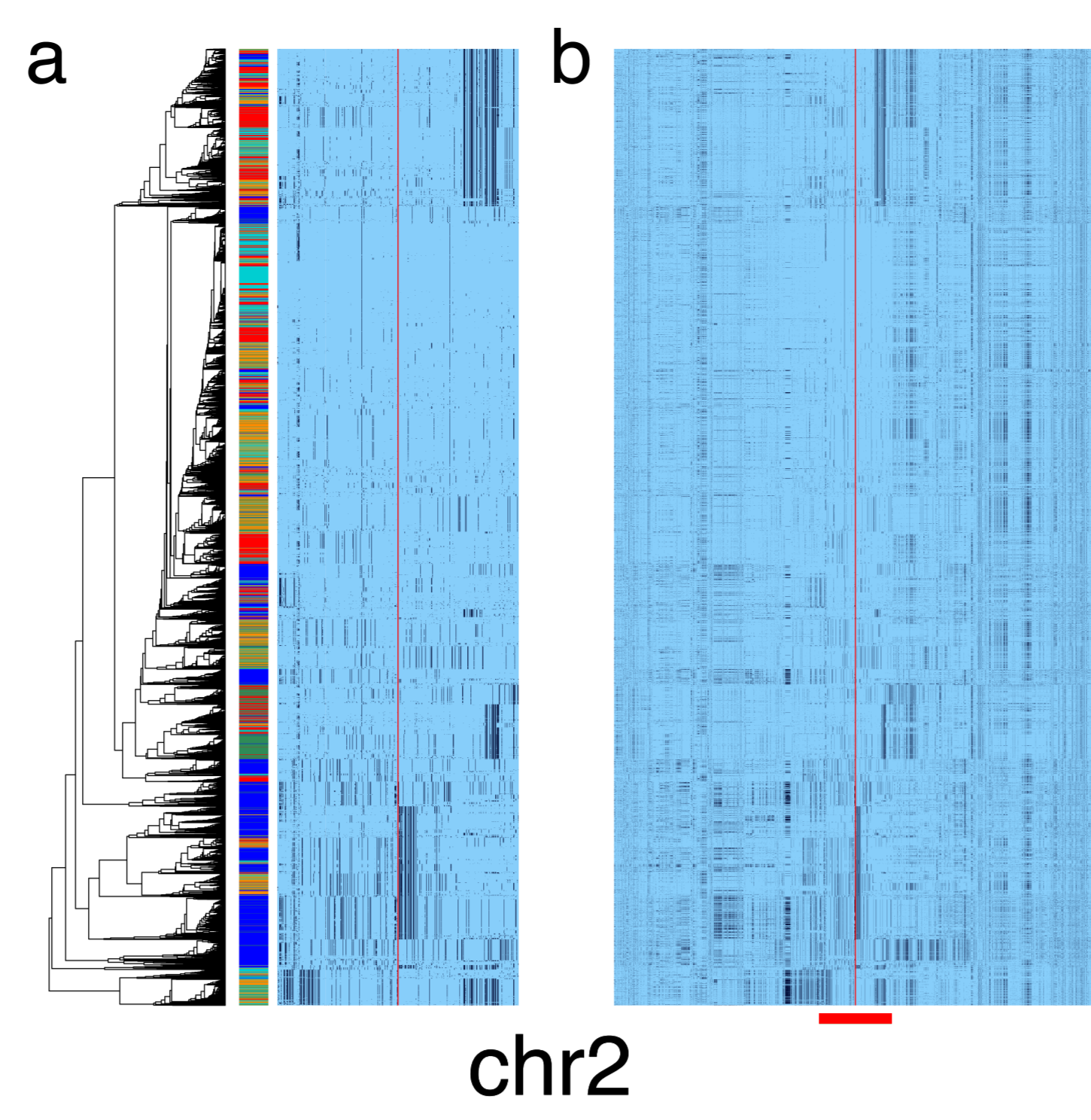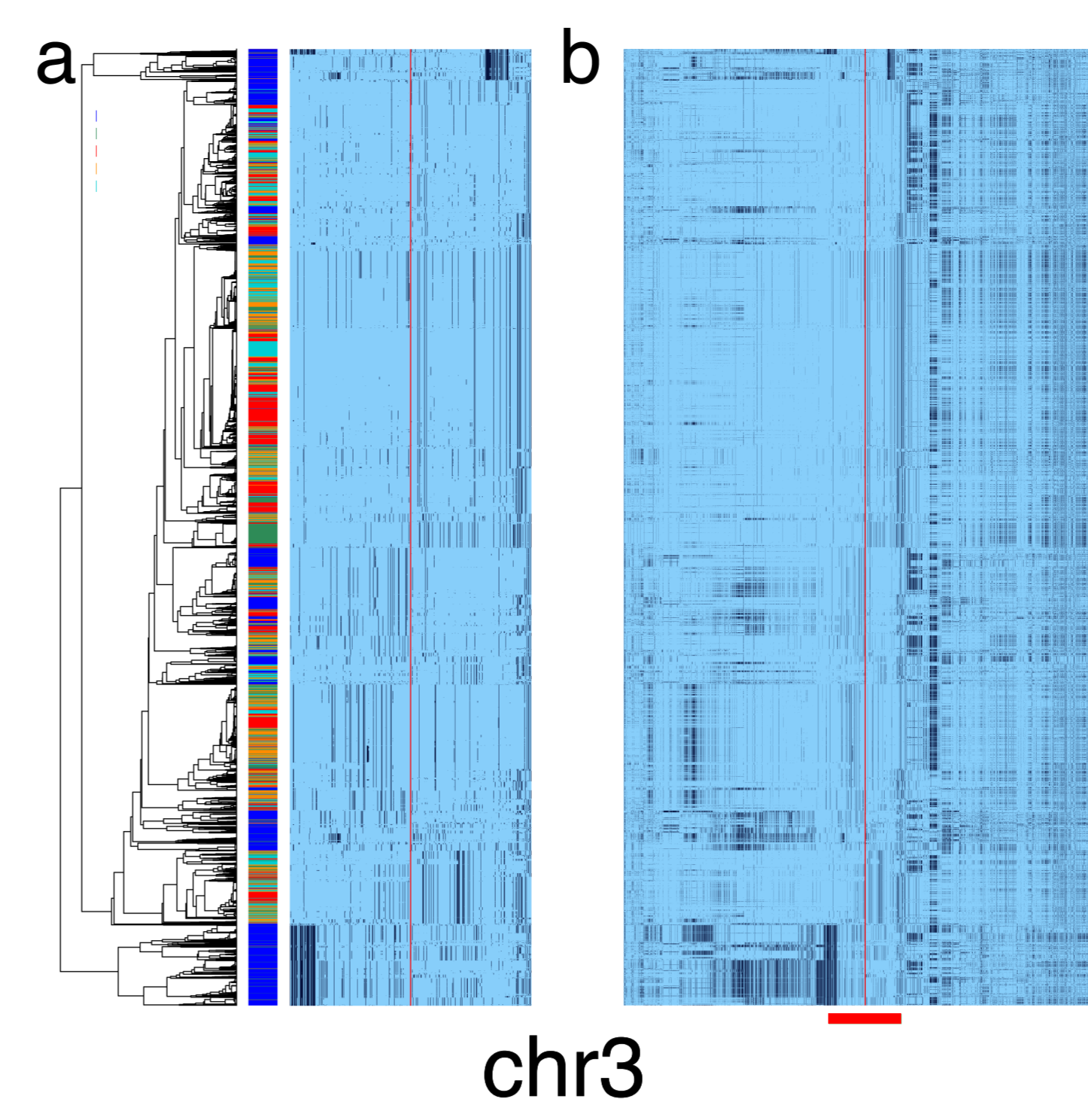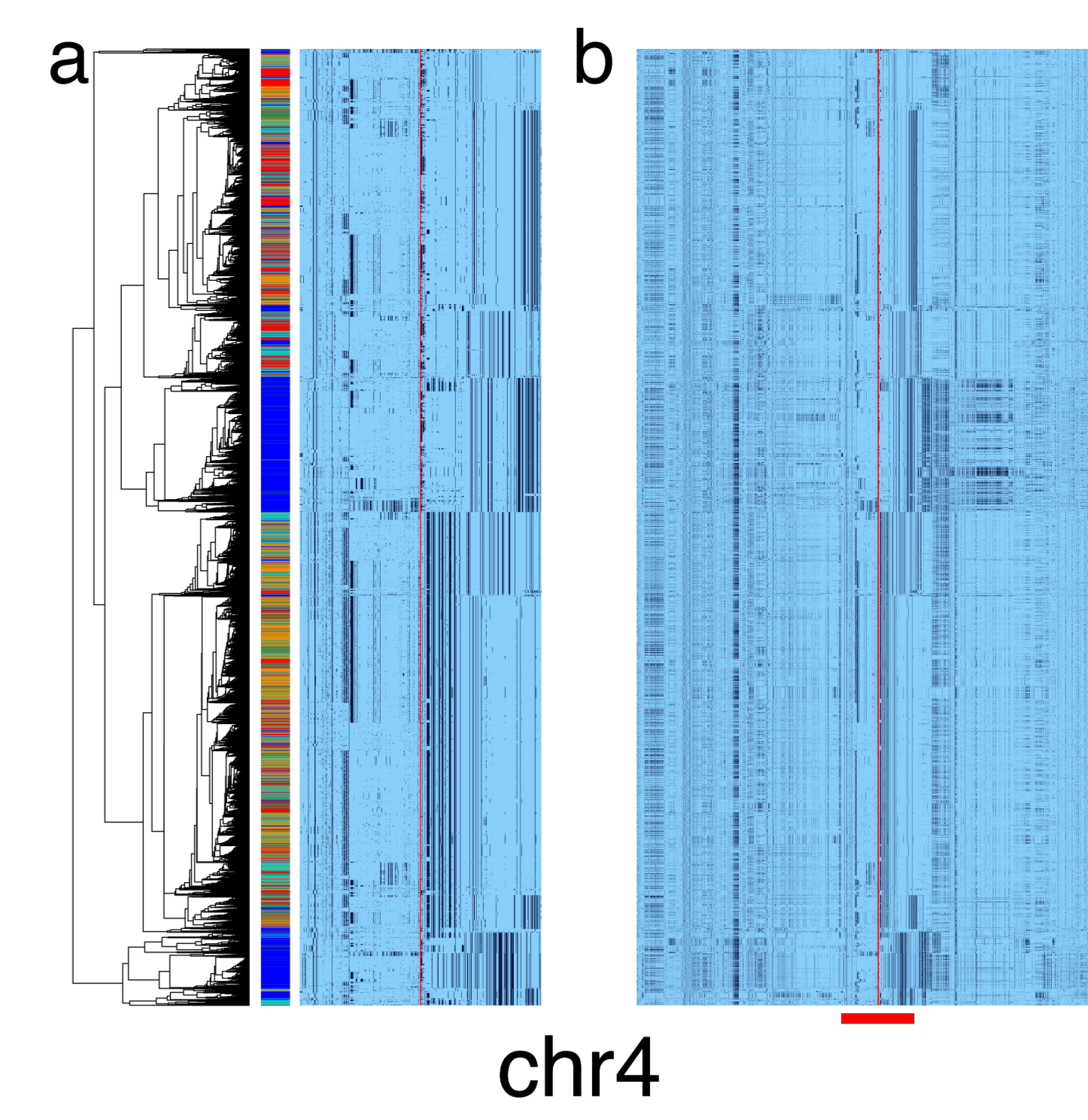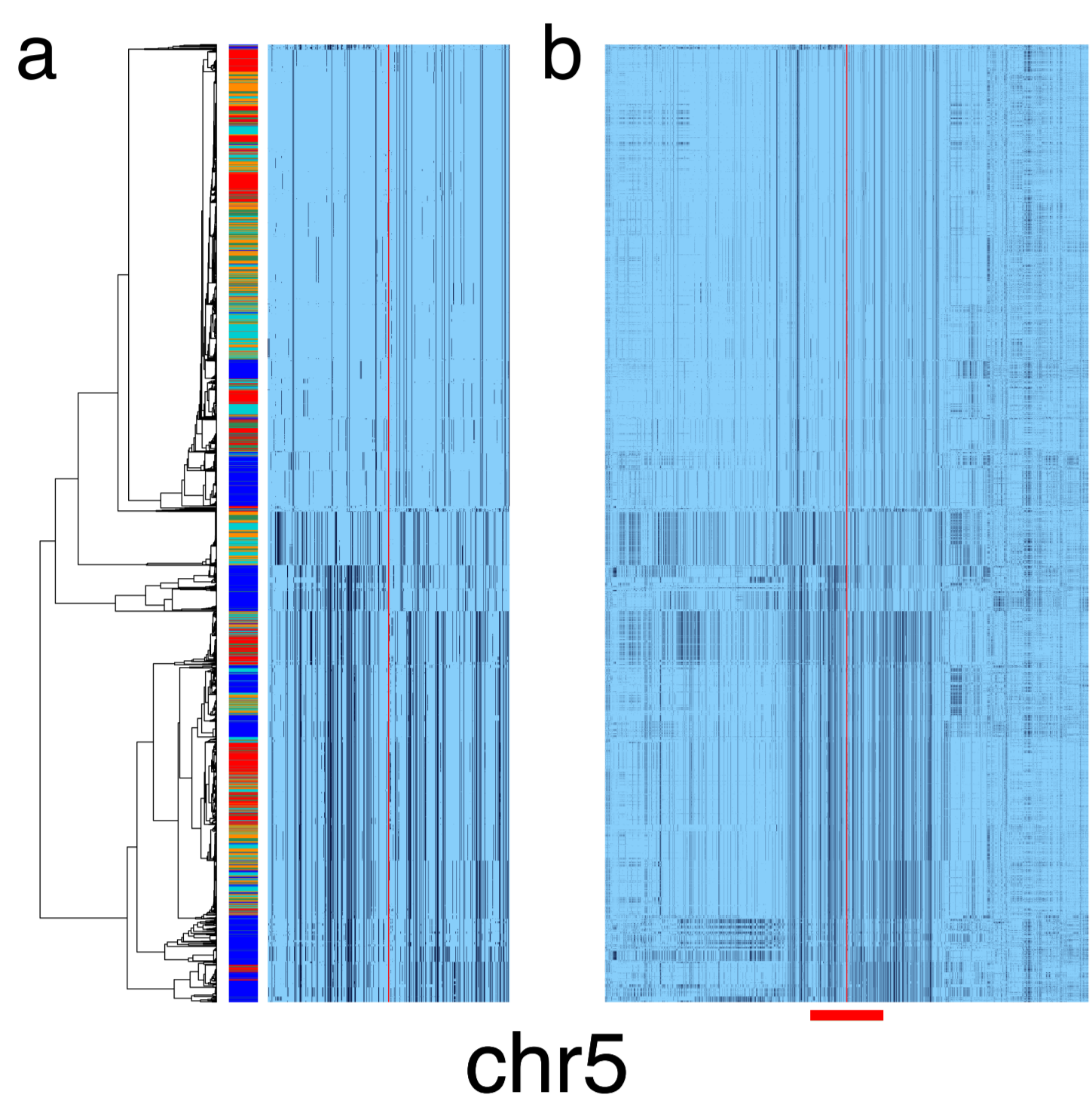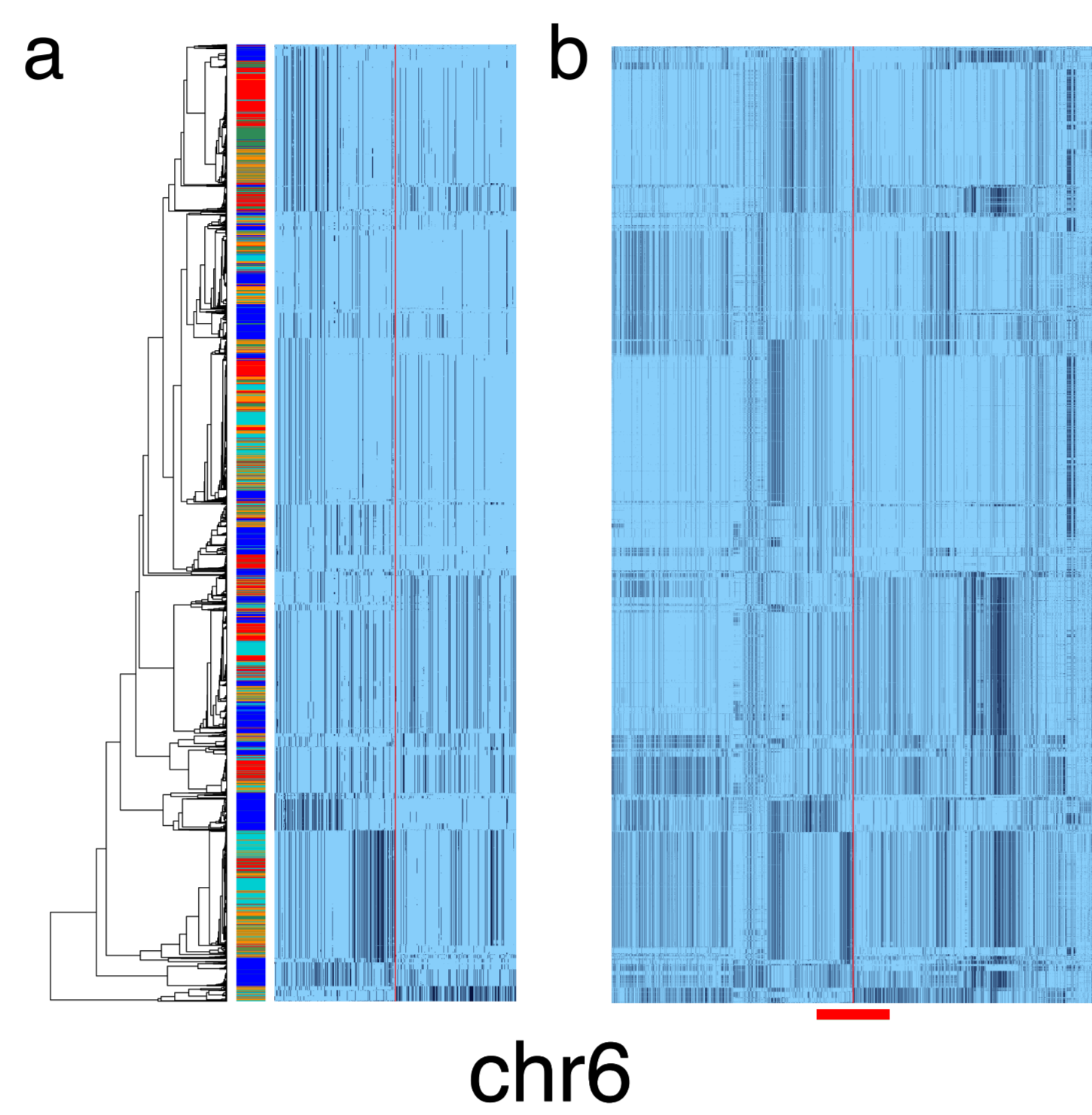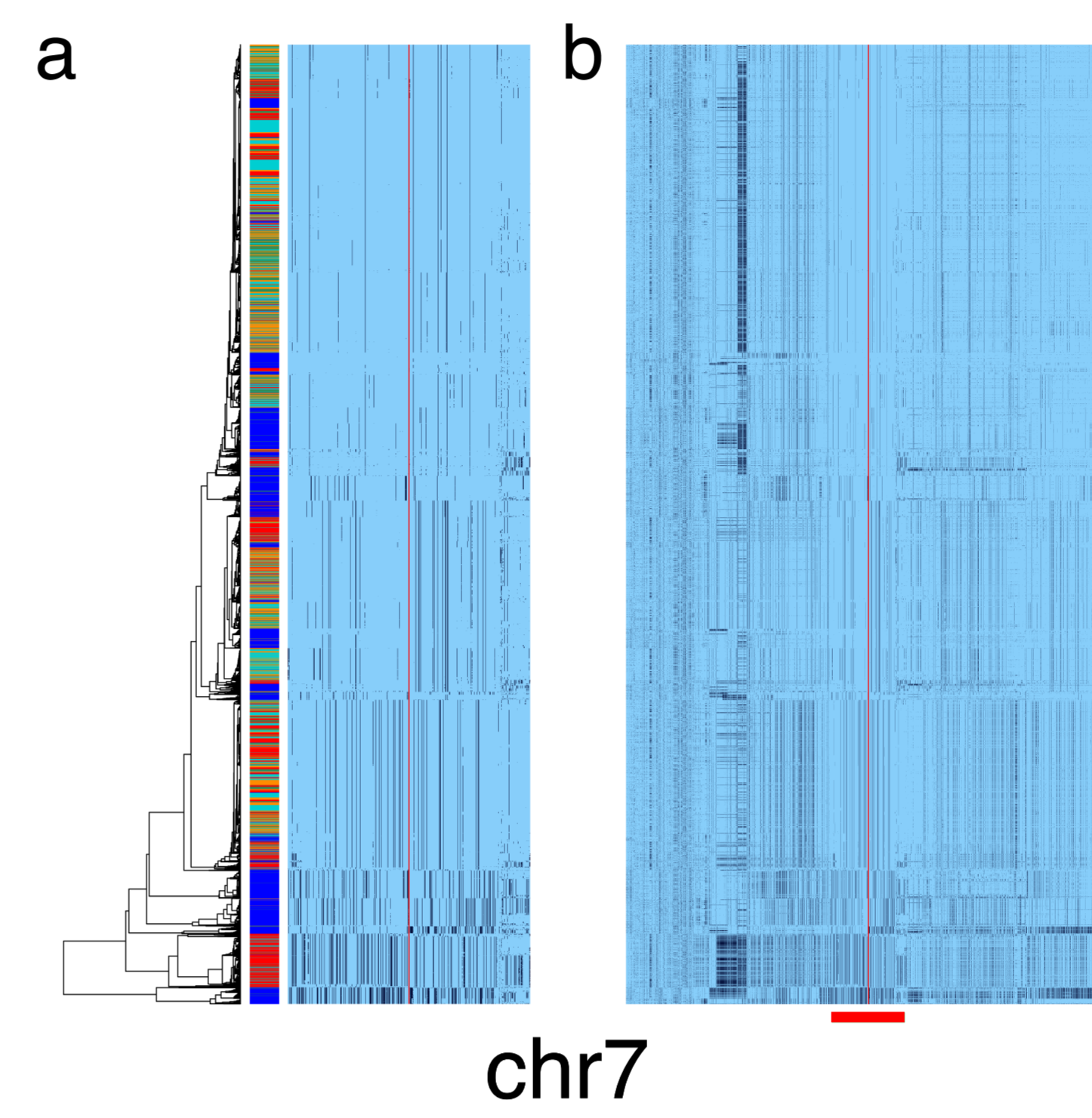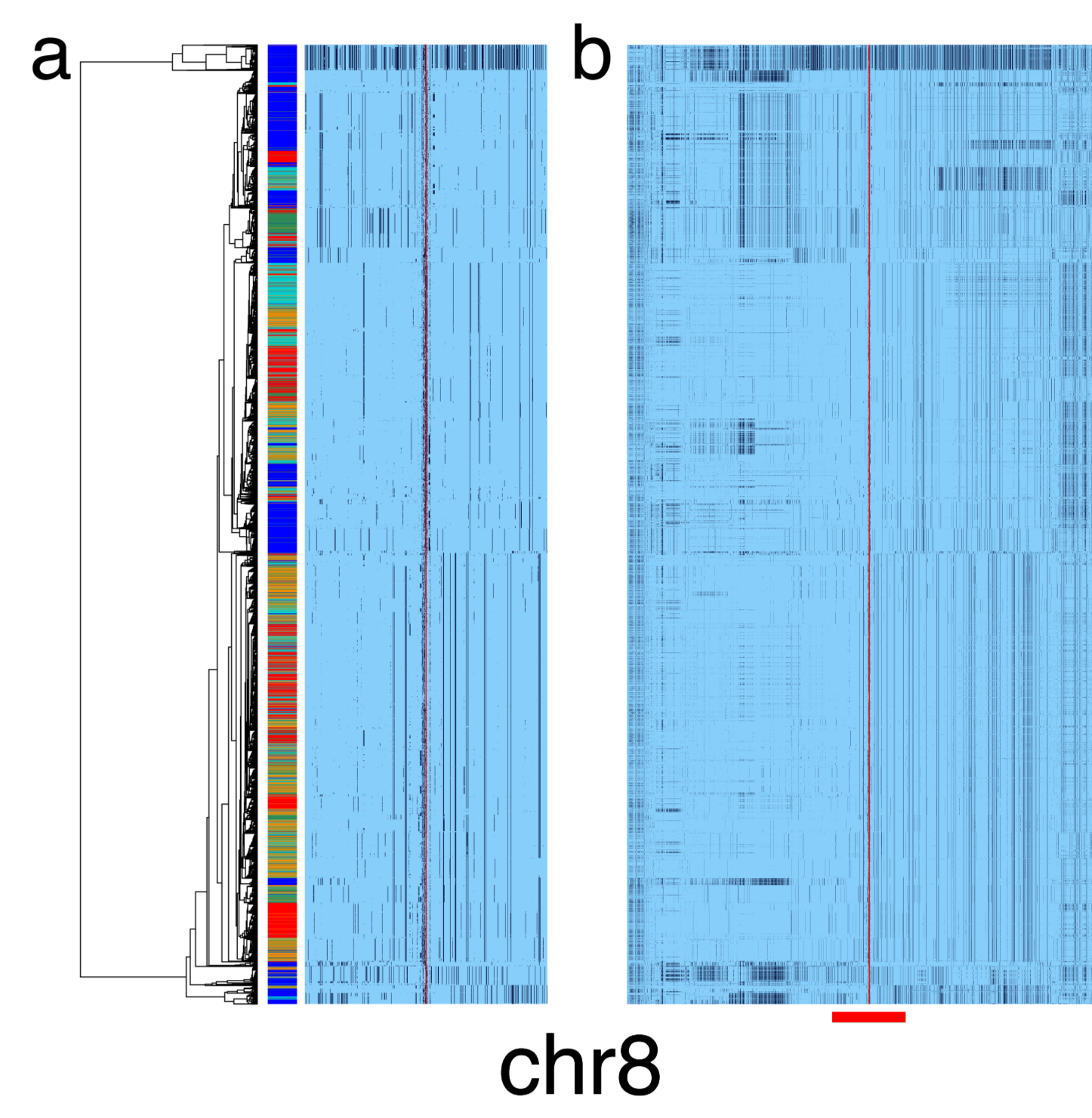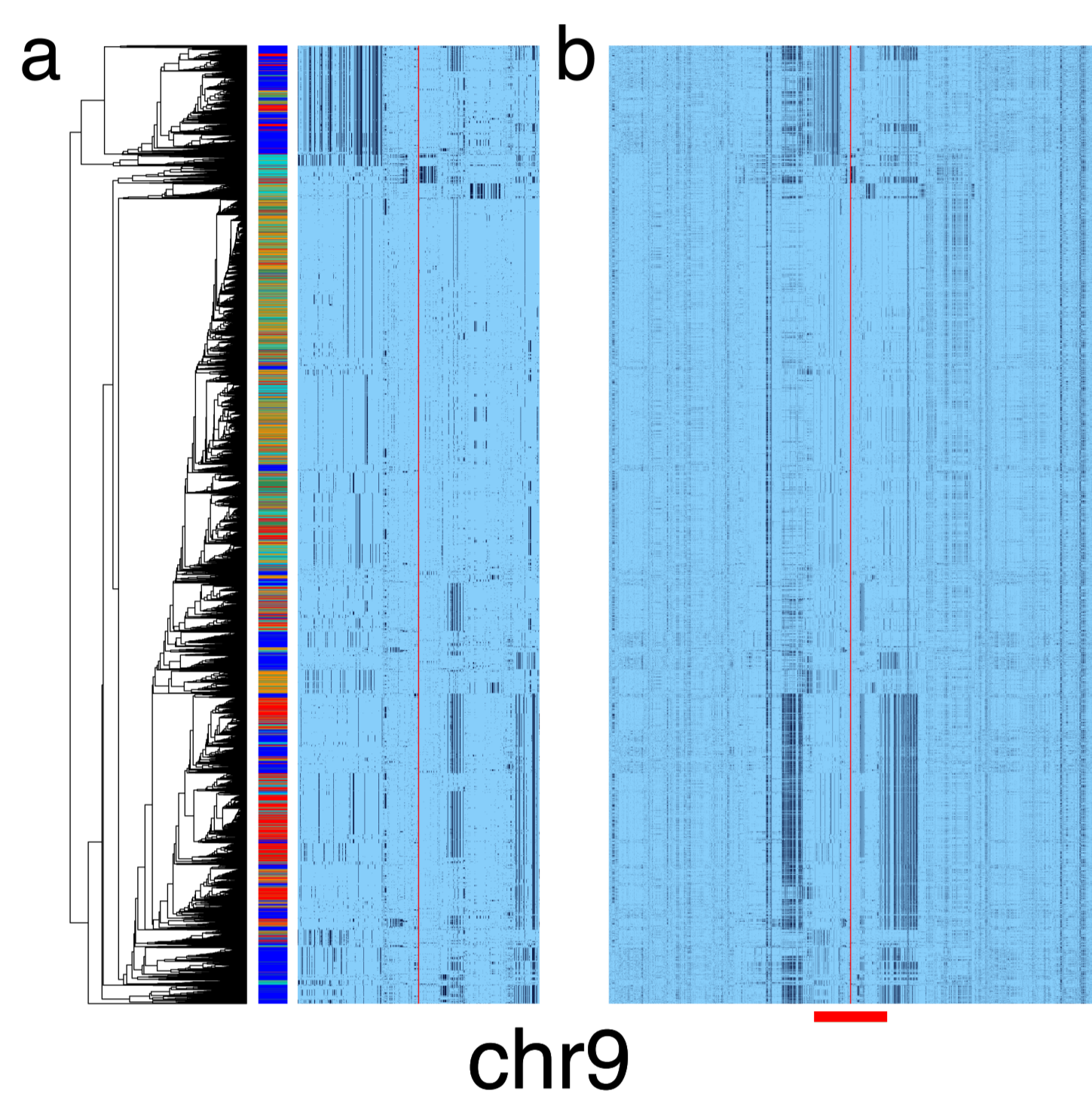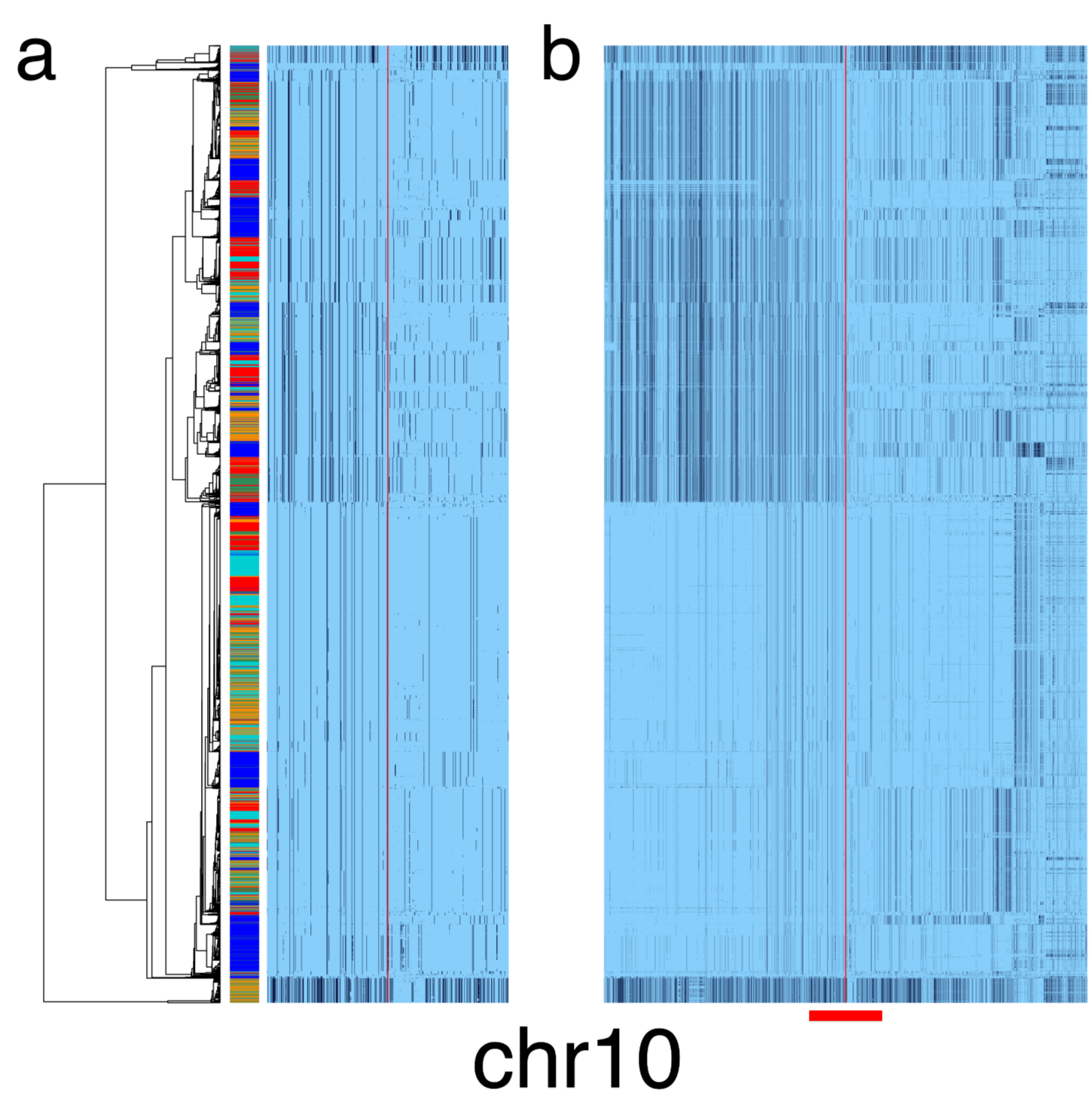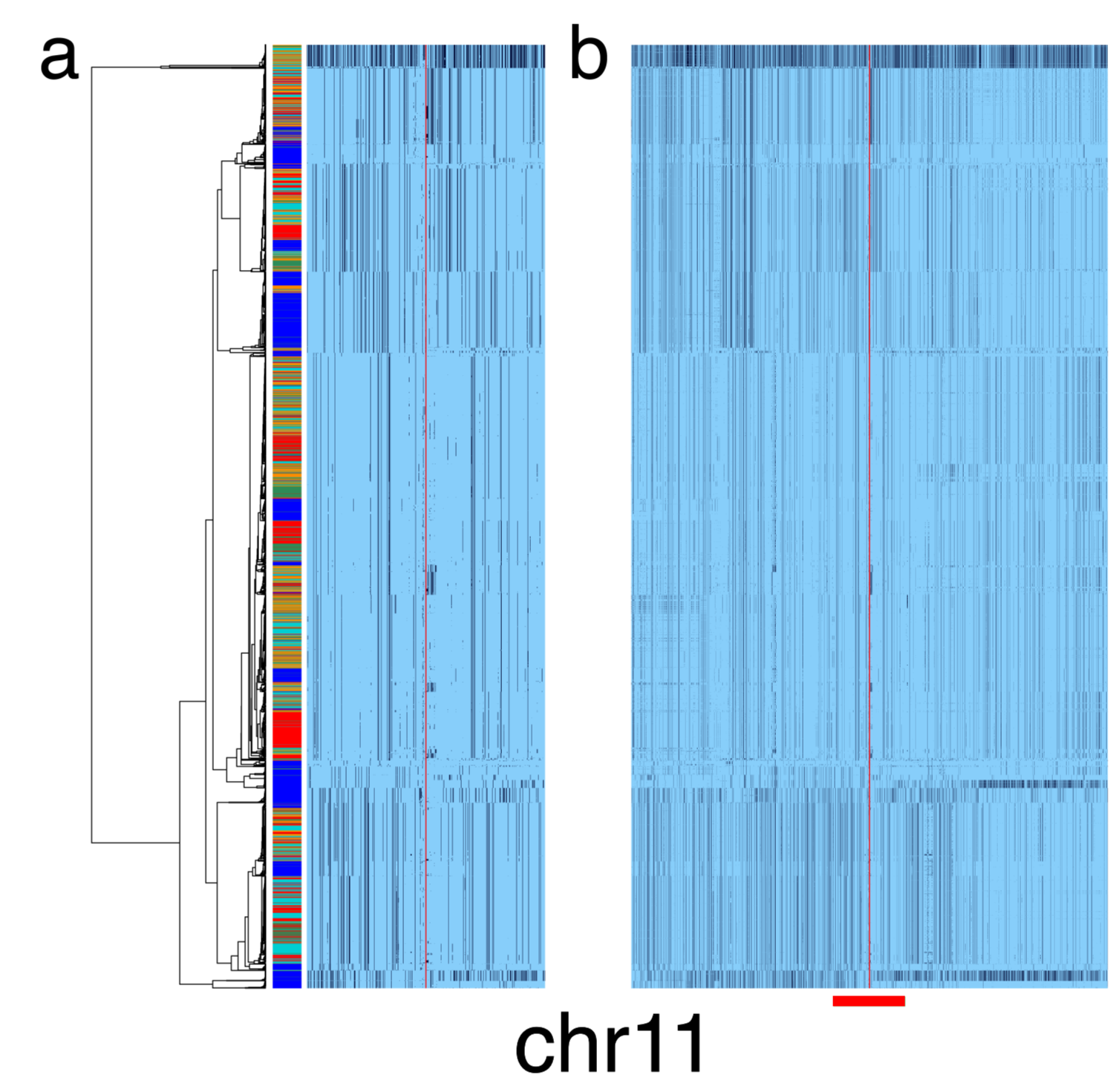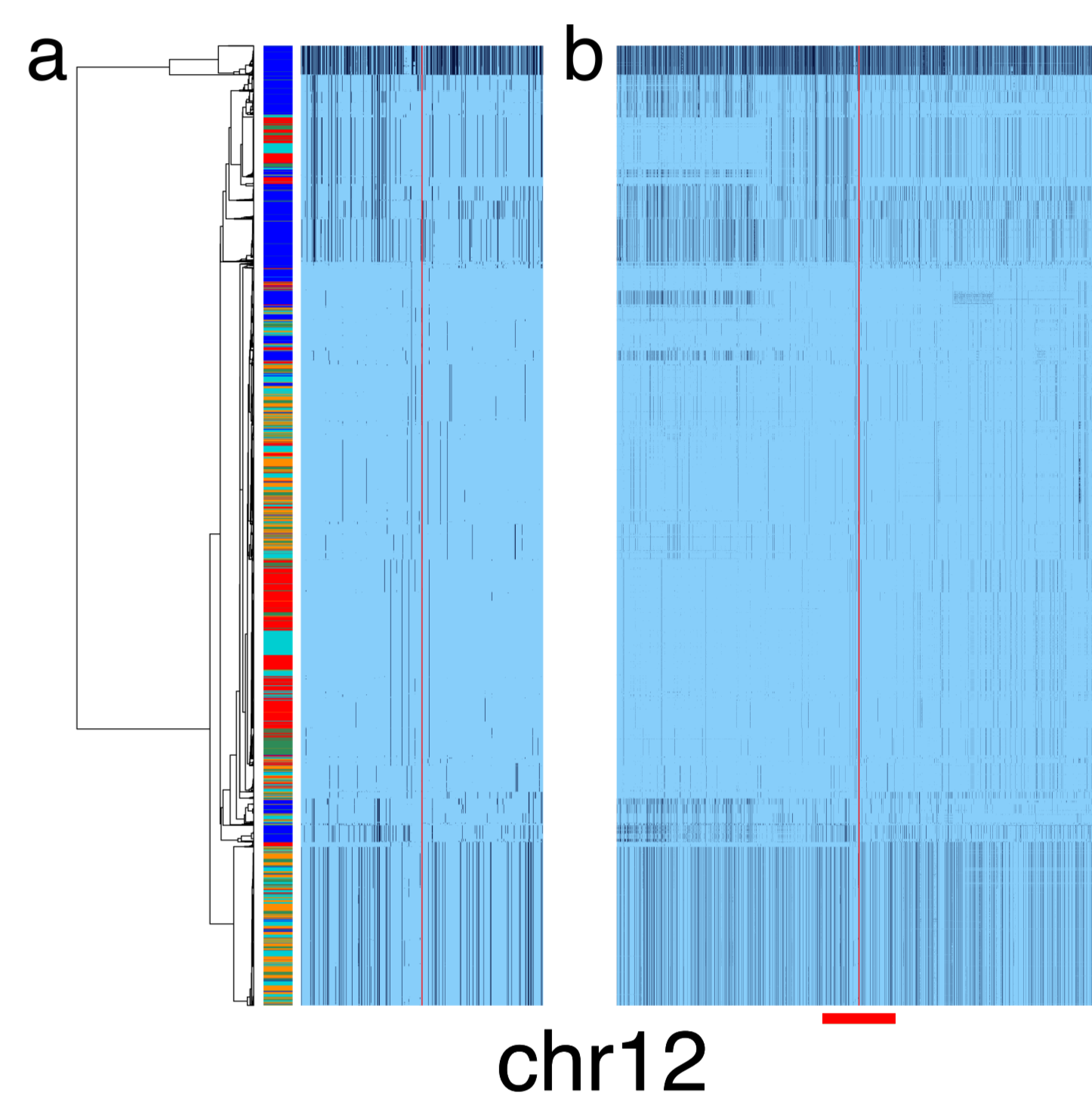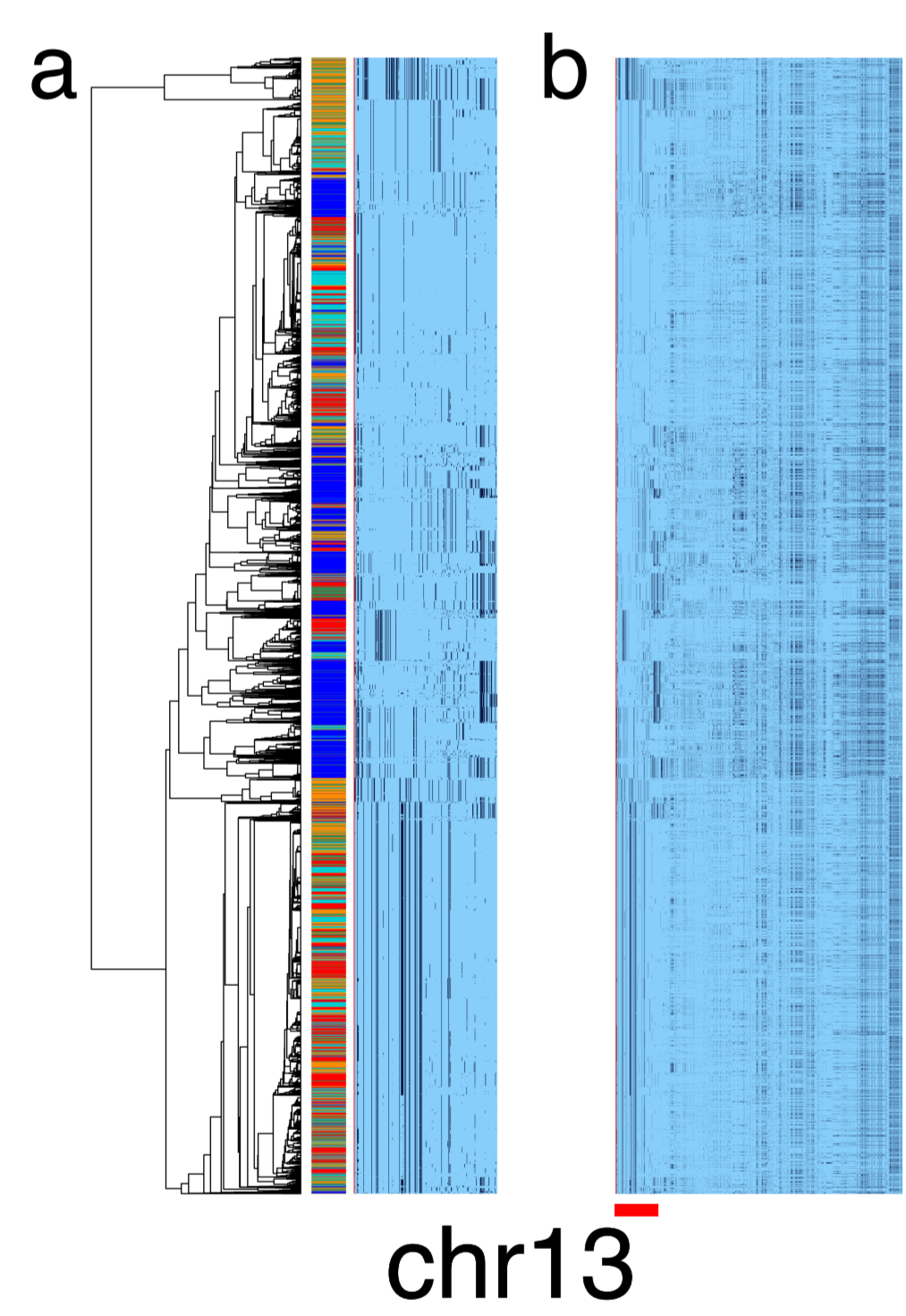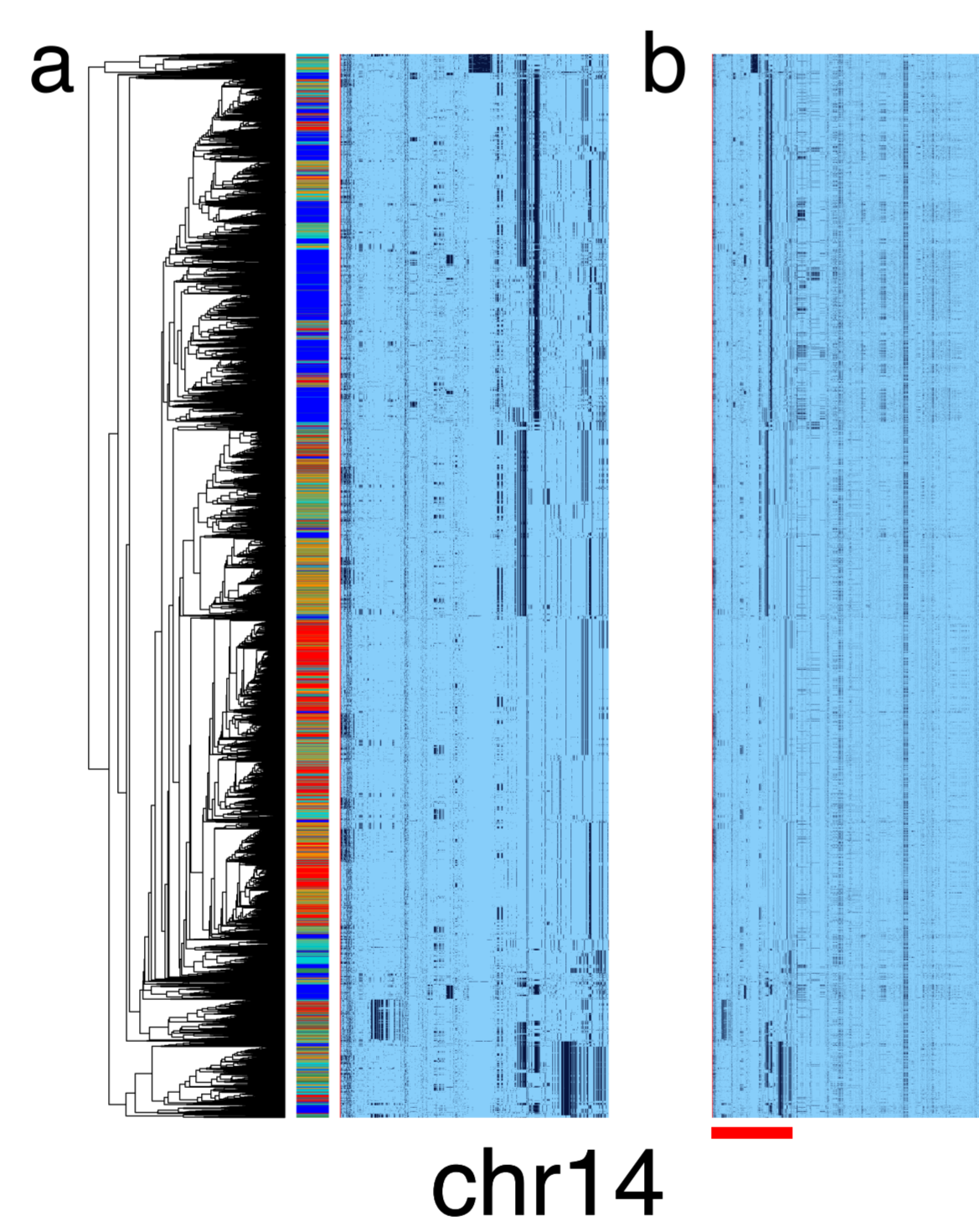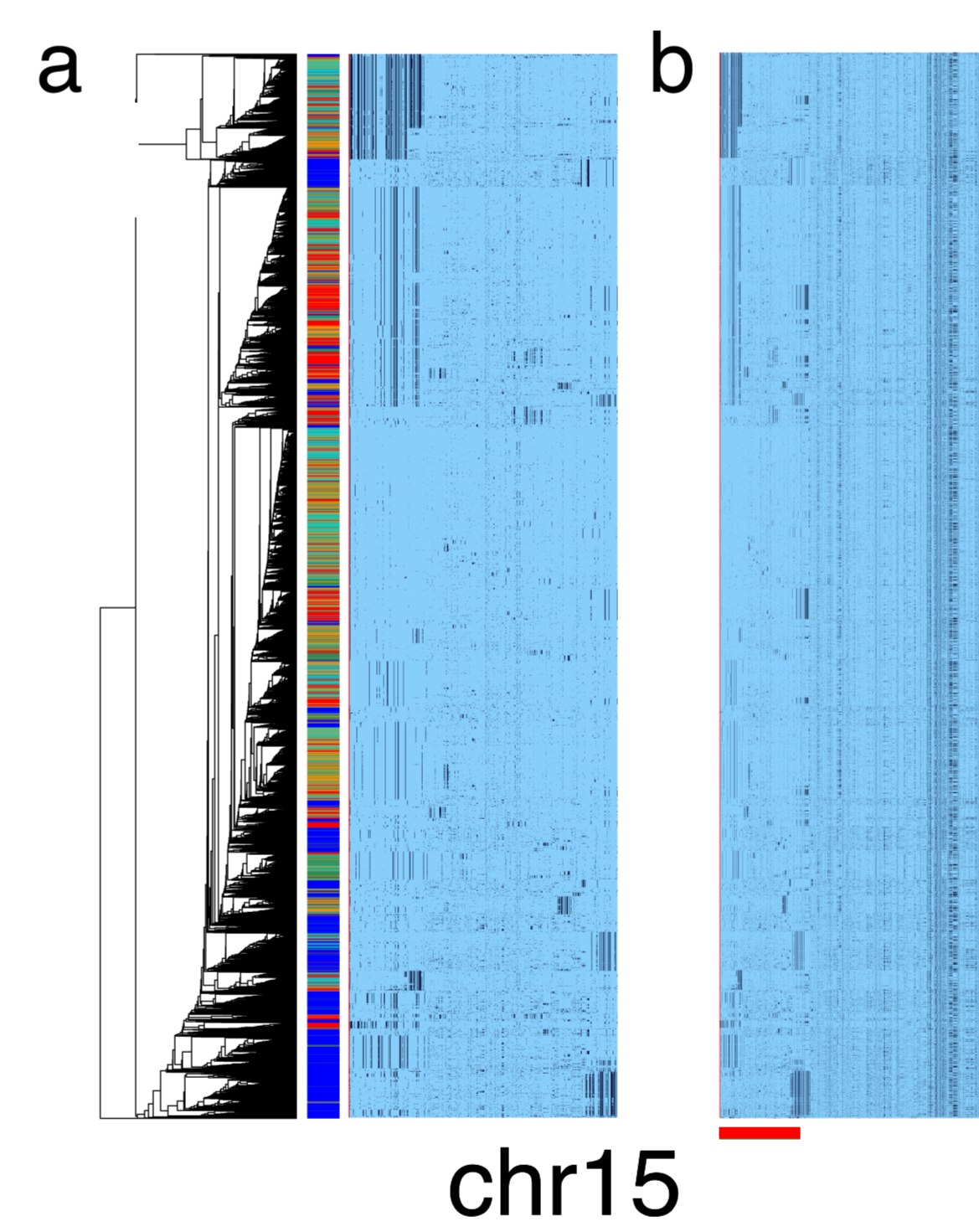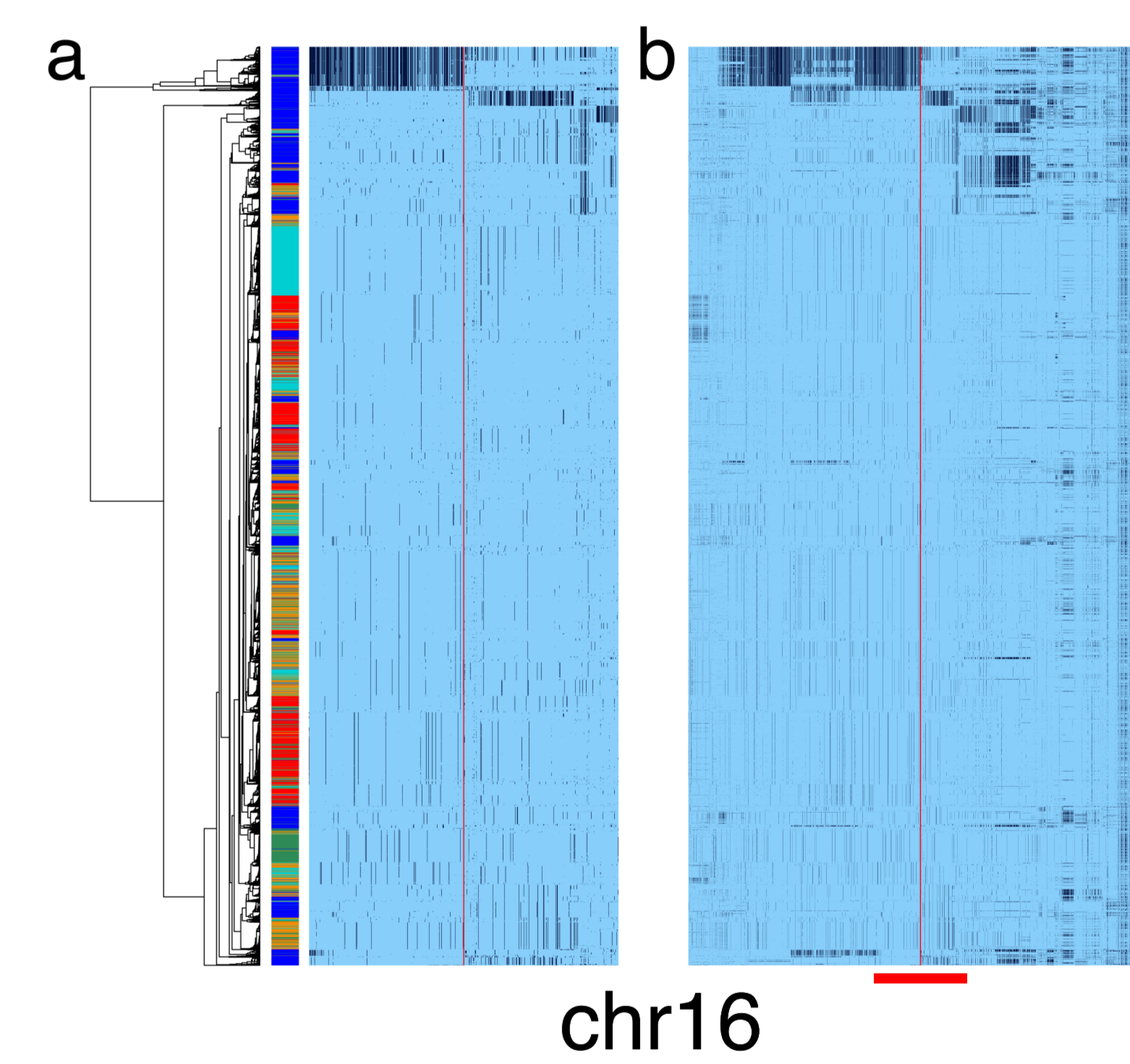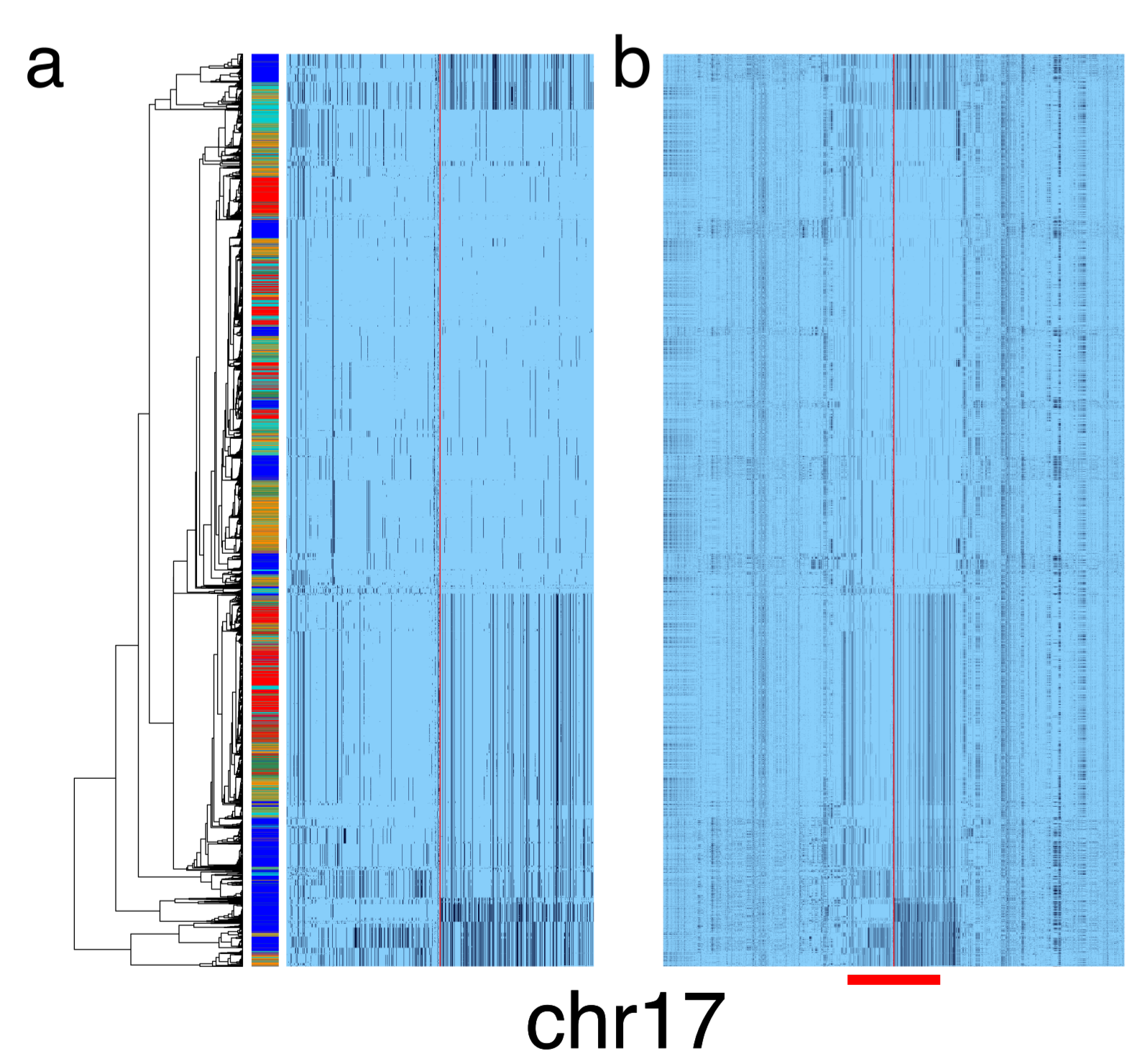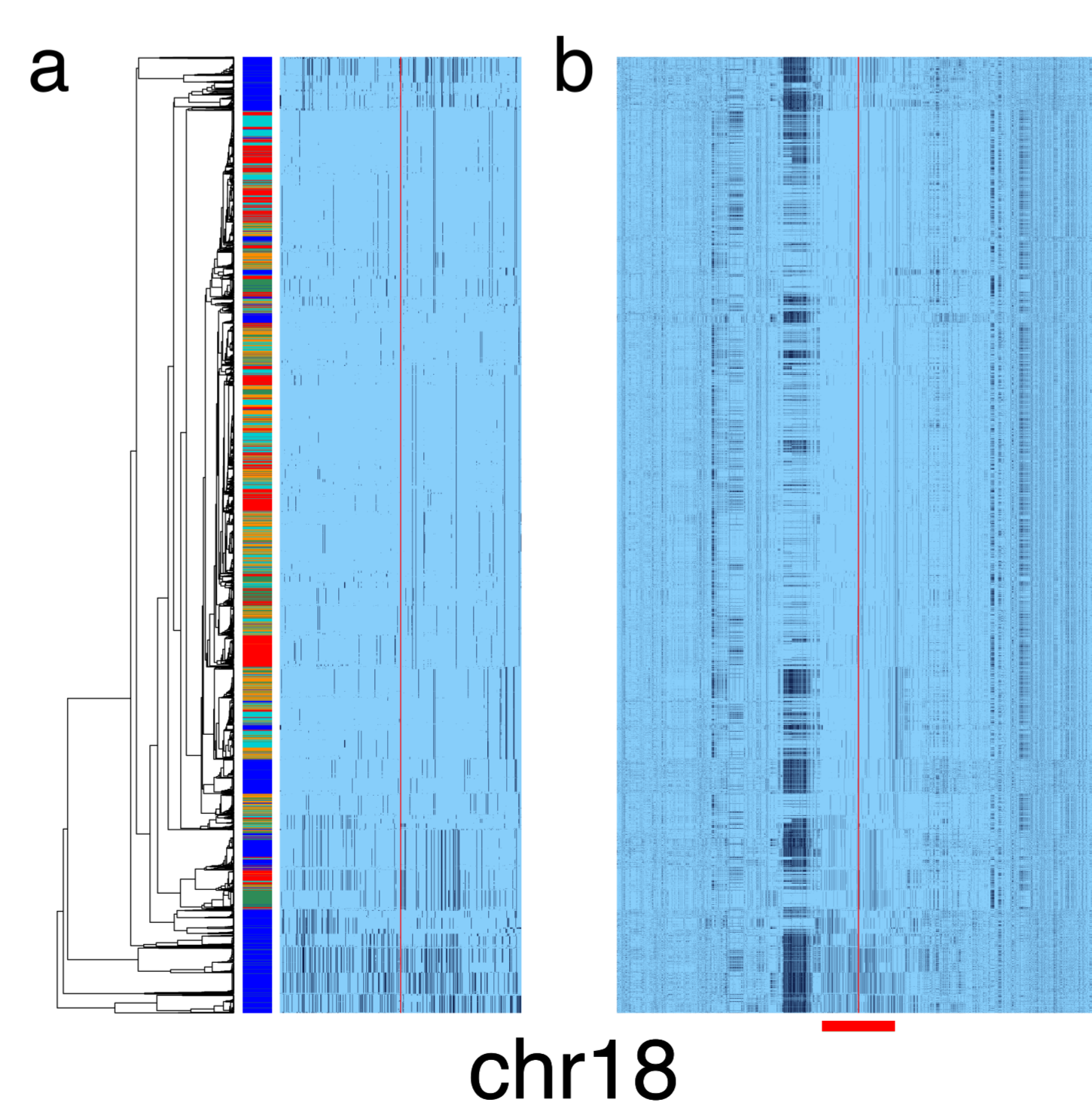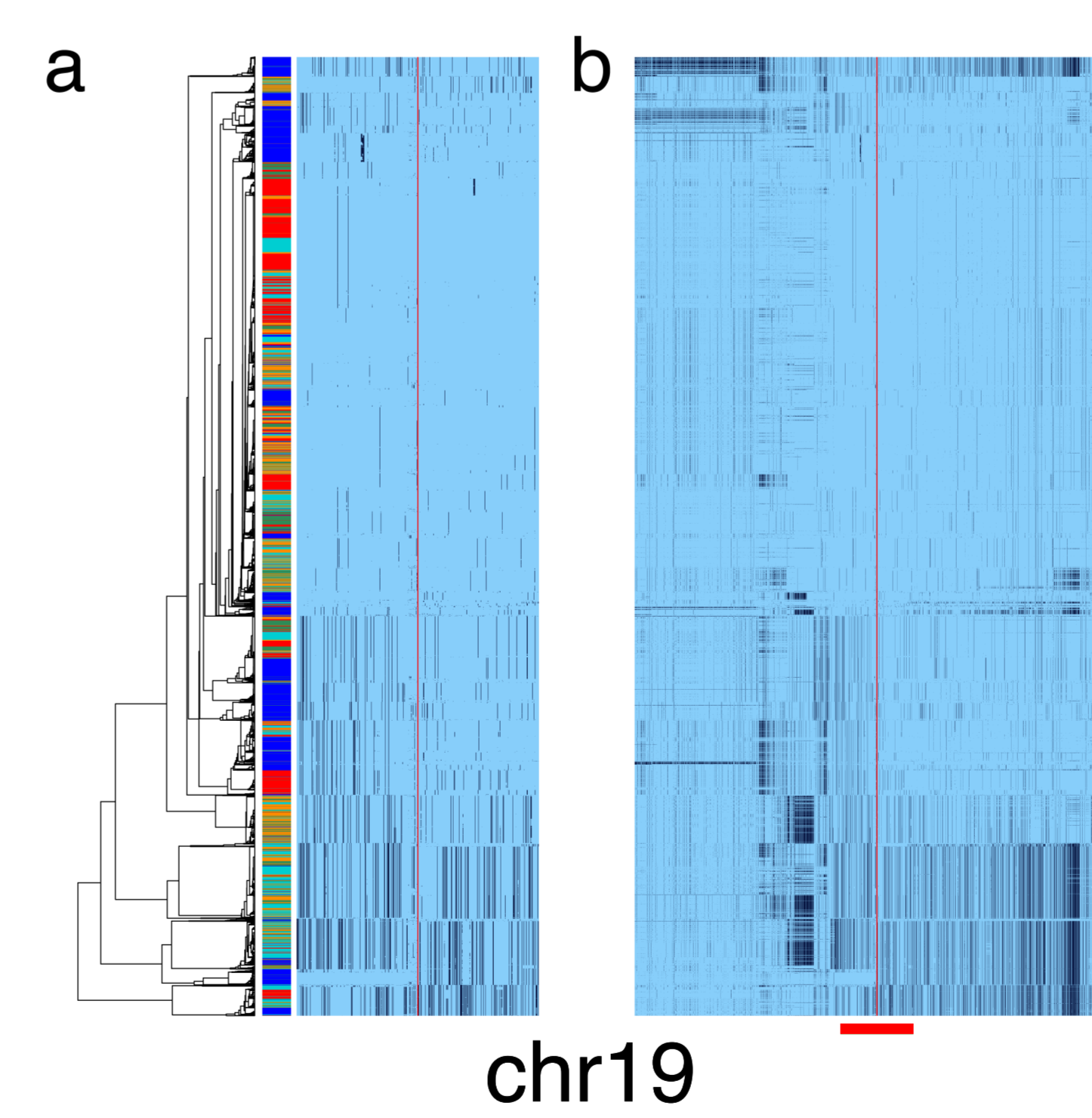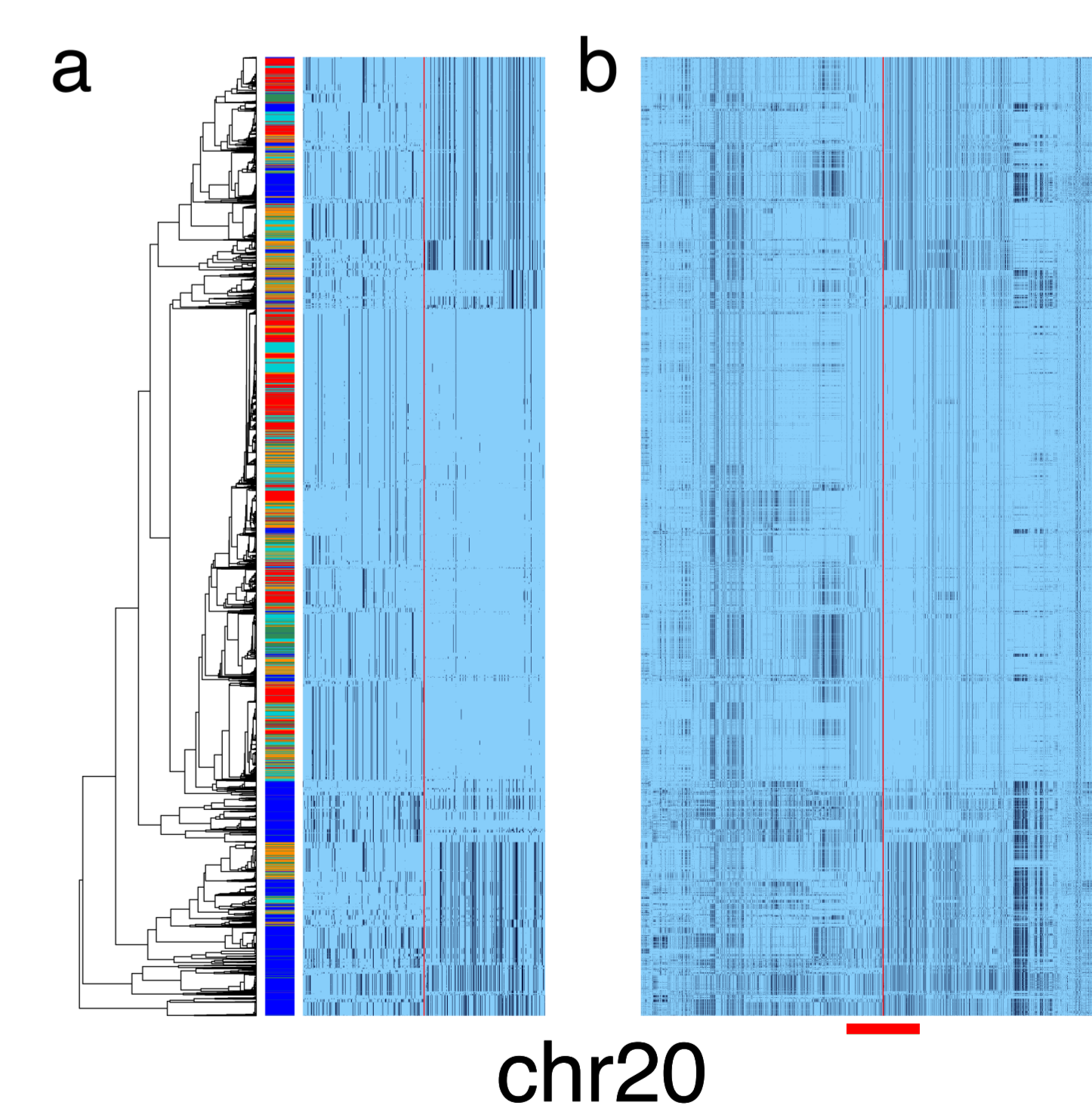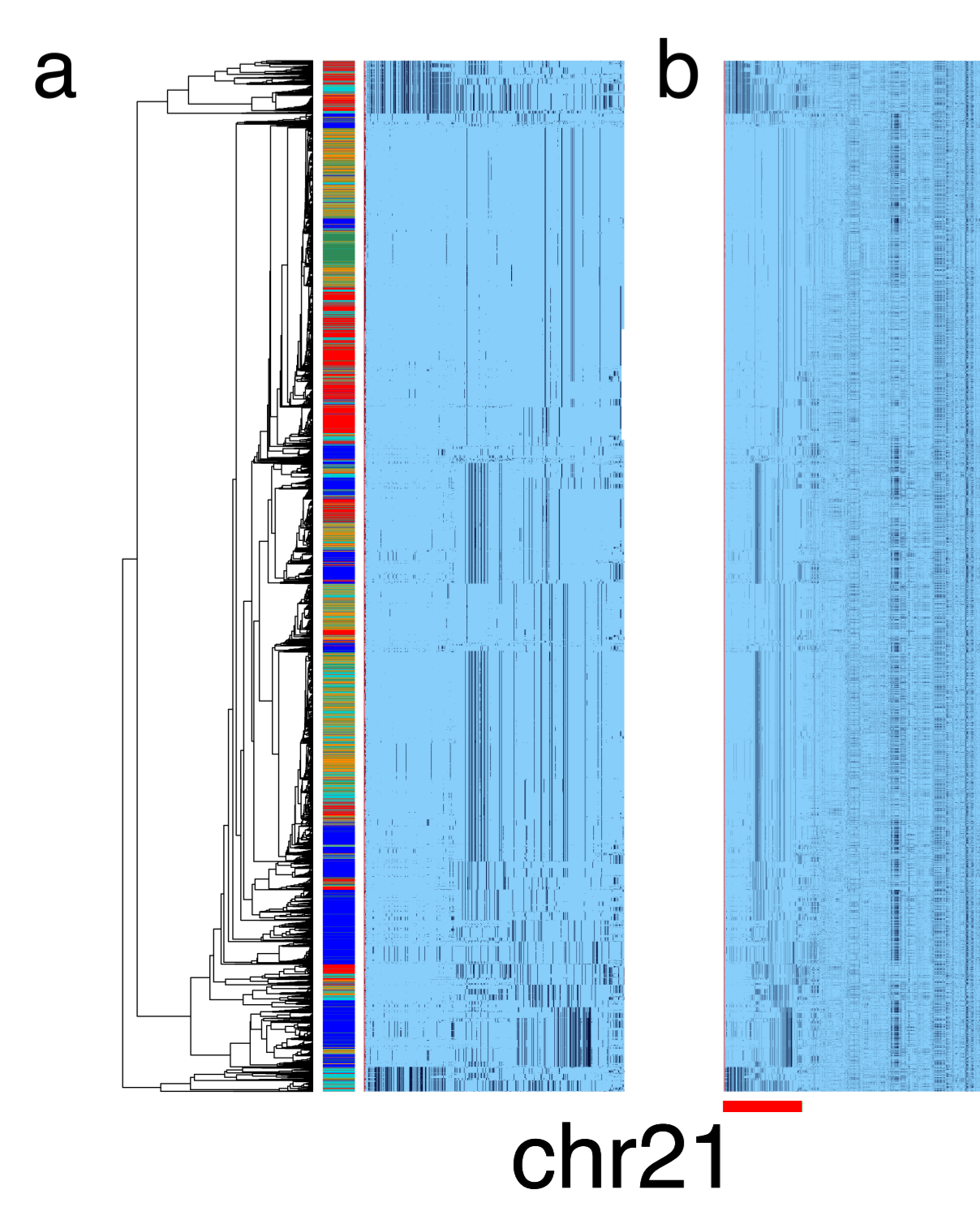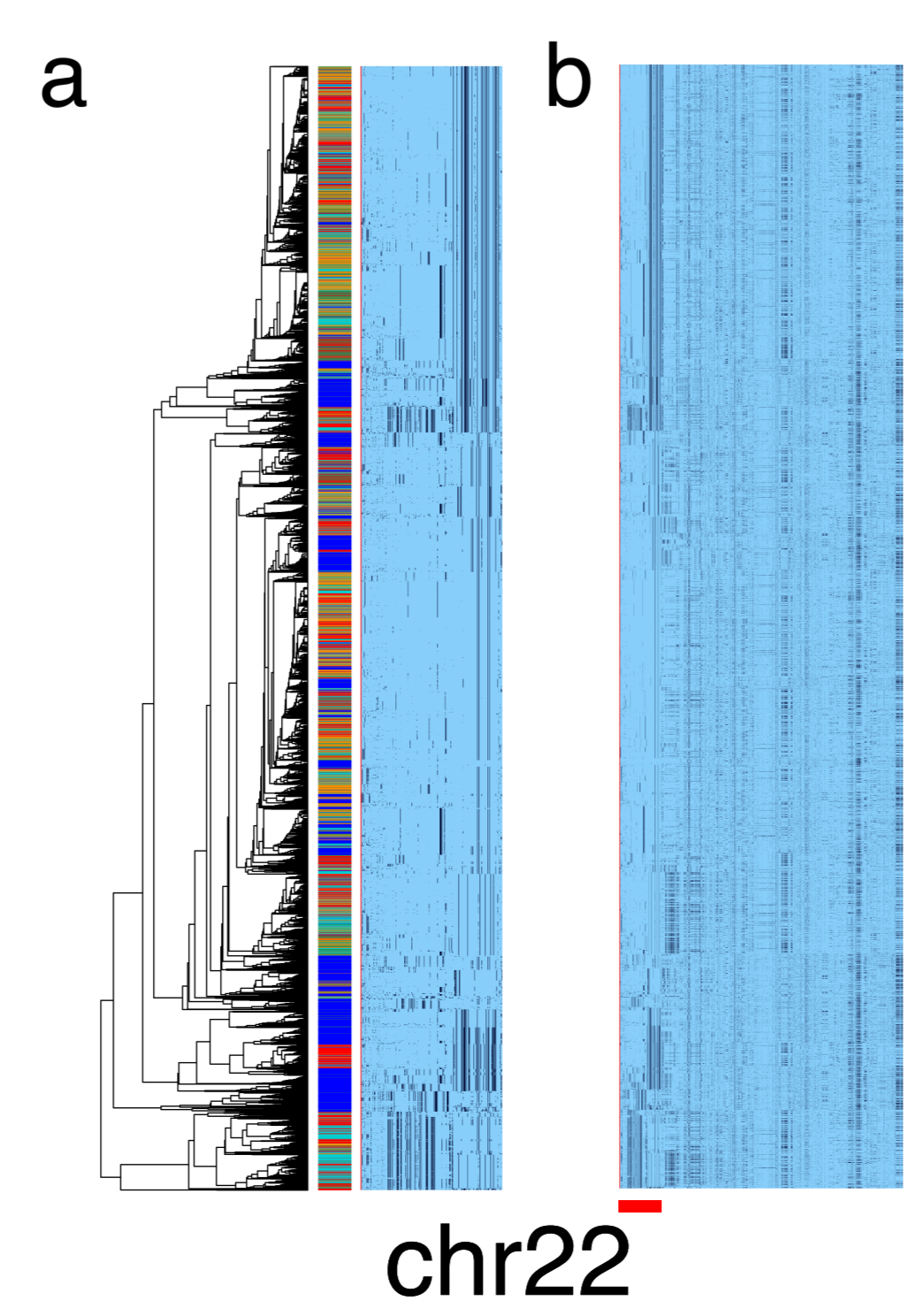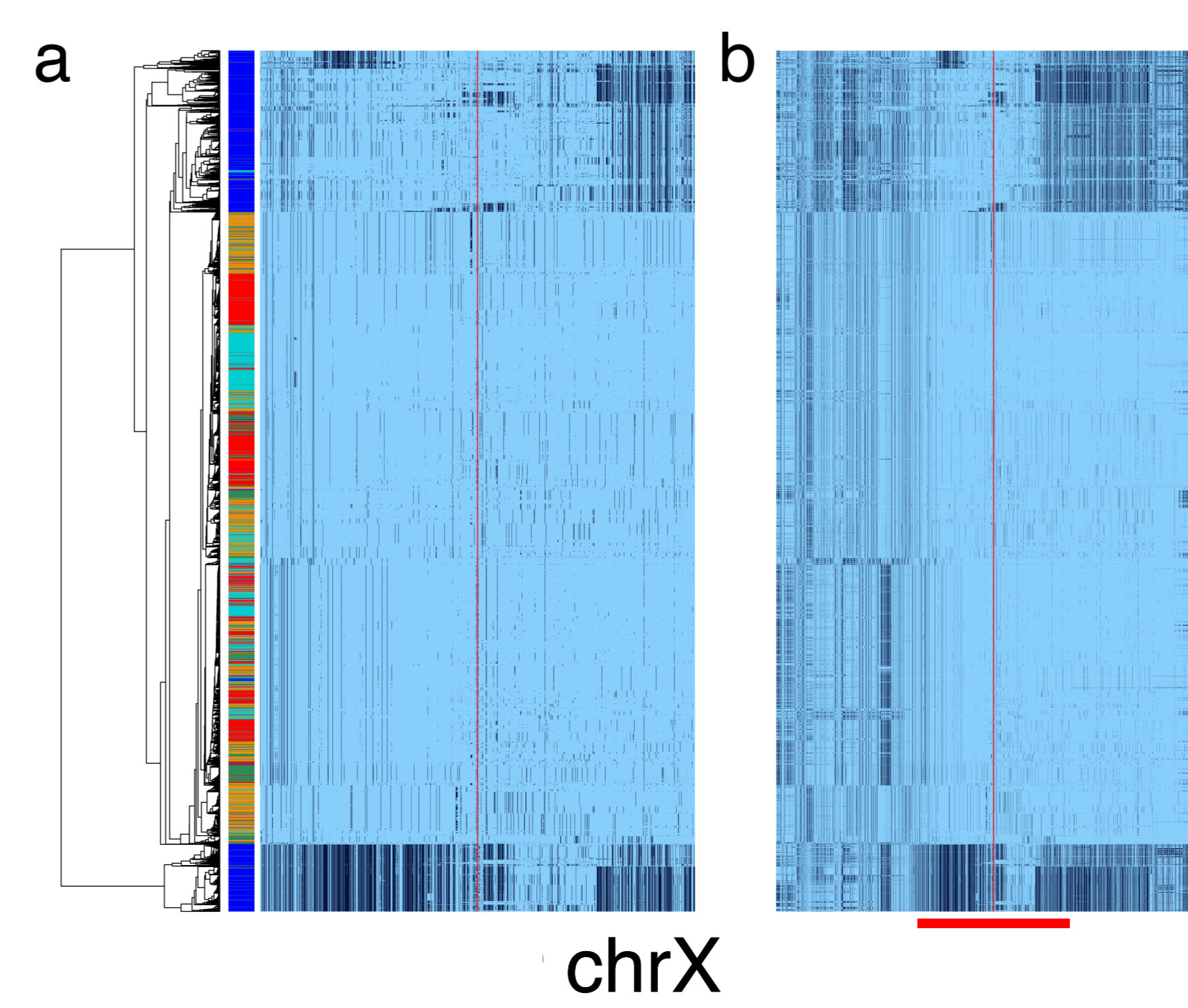

AFR  
AMR  
EAS  
EUR  
SAS

Supplement: Figure 2—source data 2. [file elife-42989-fig2-data2.pdf]

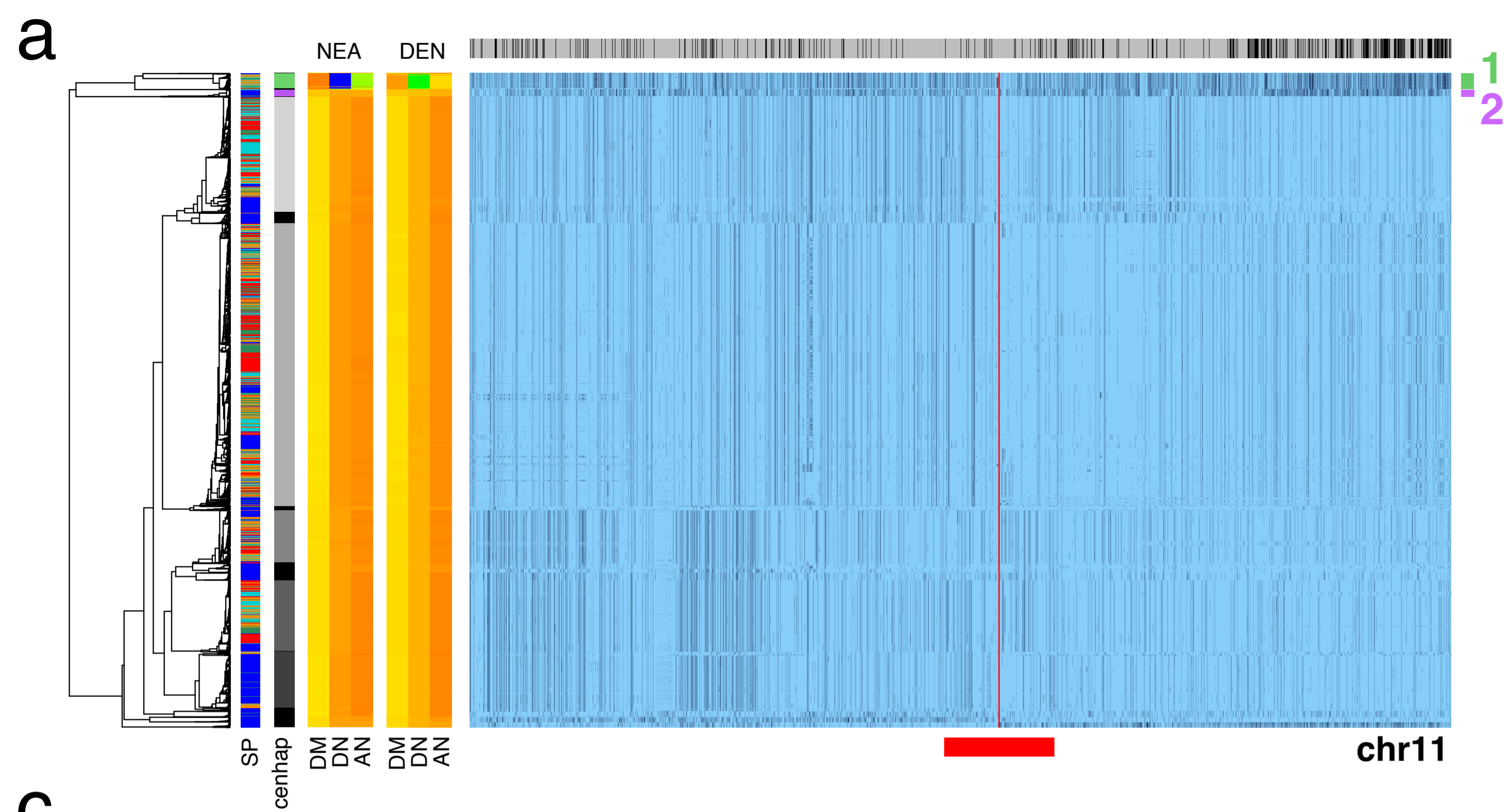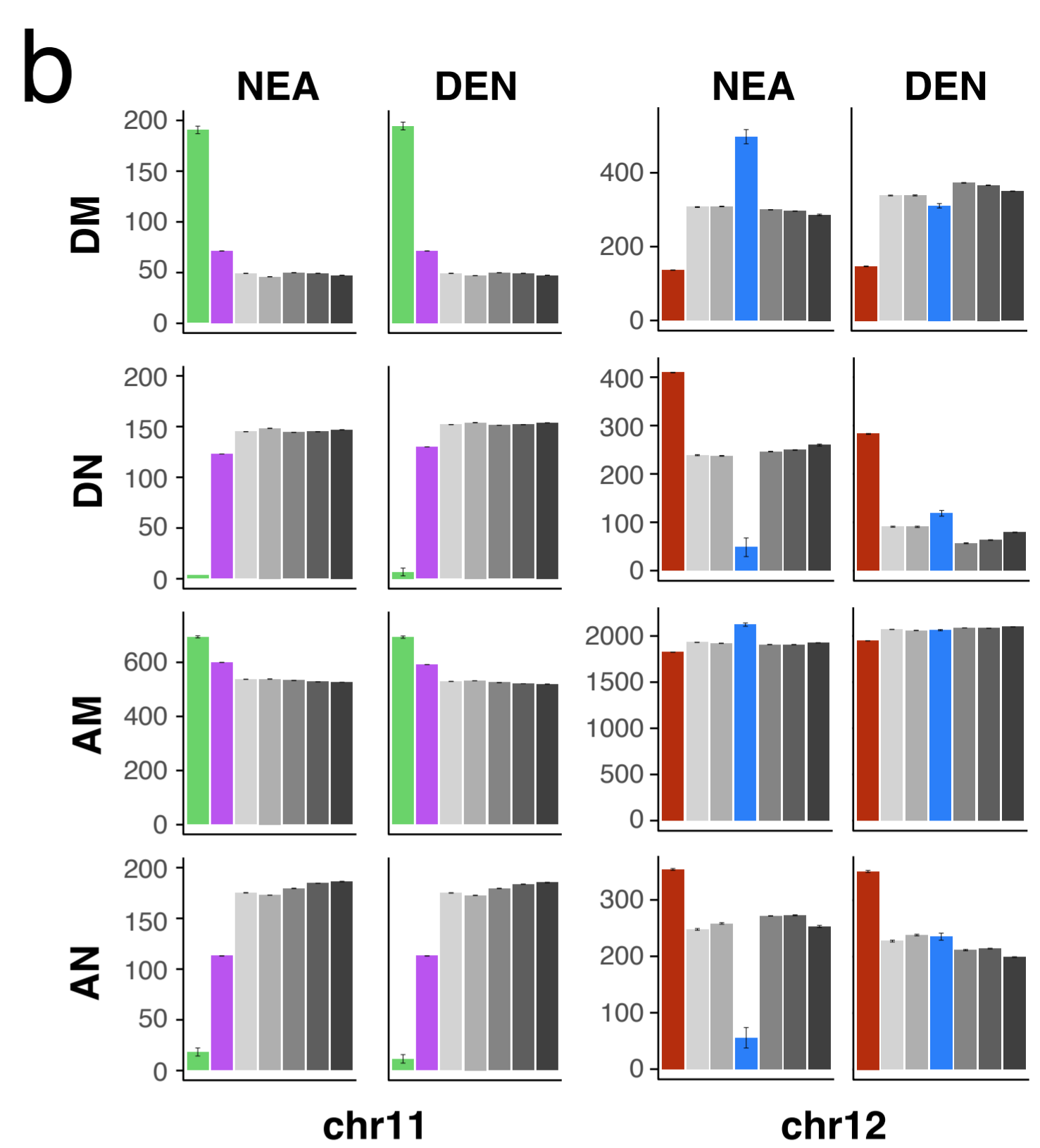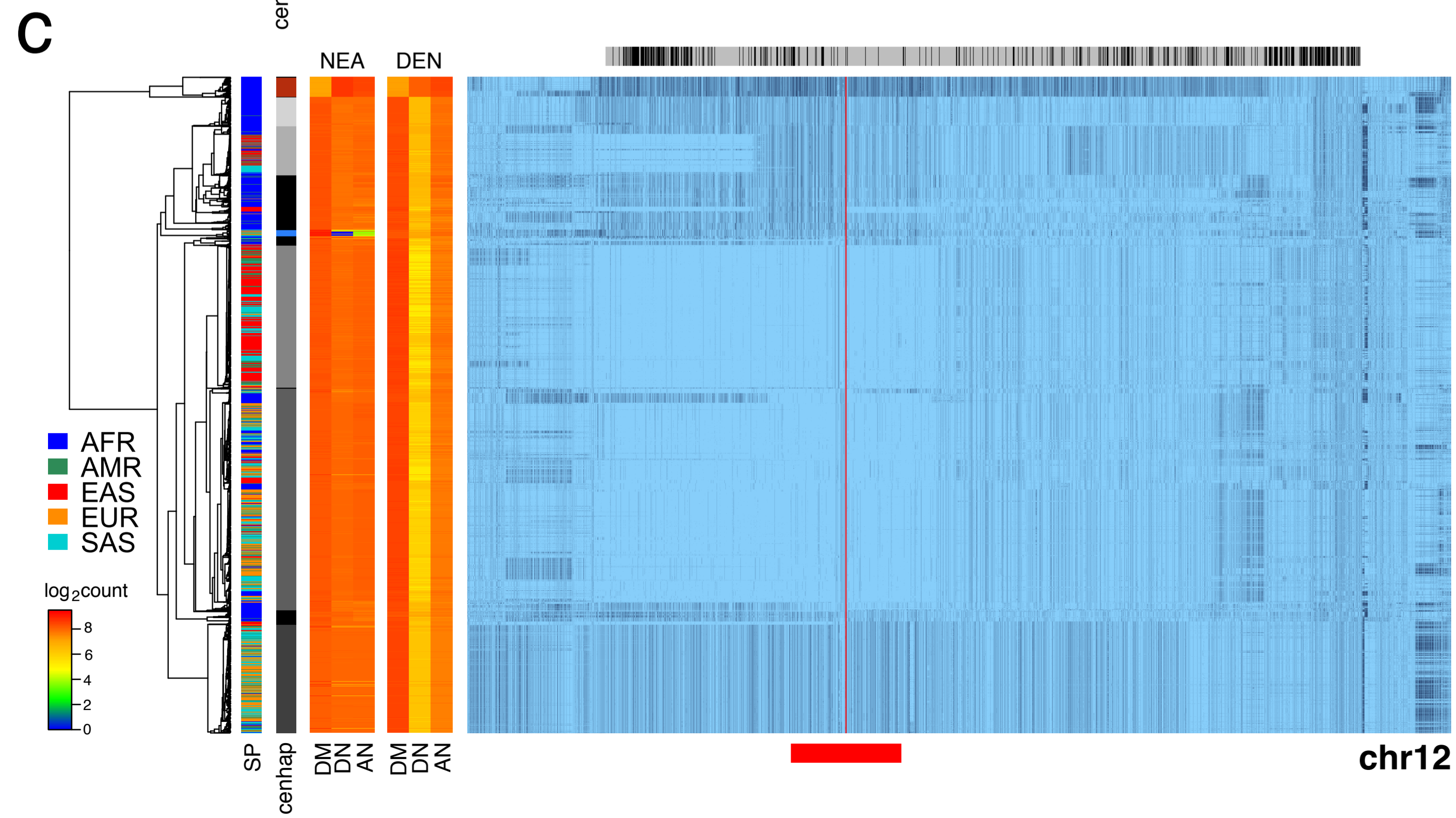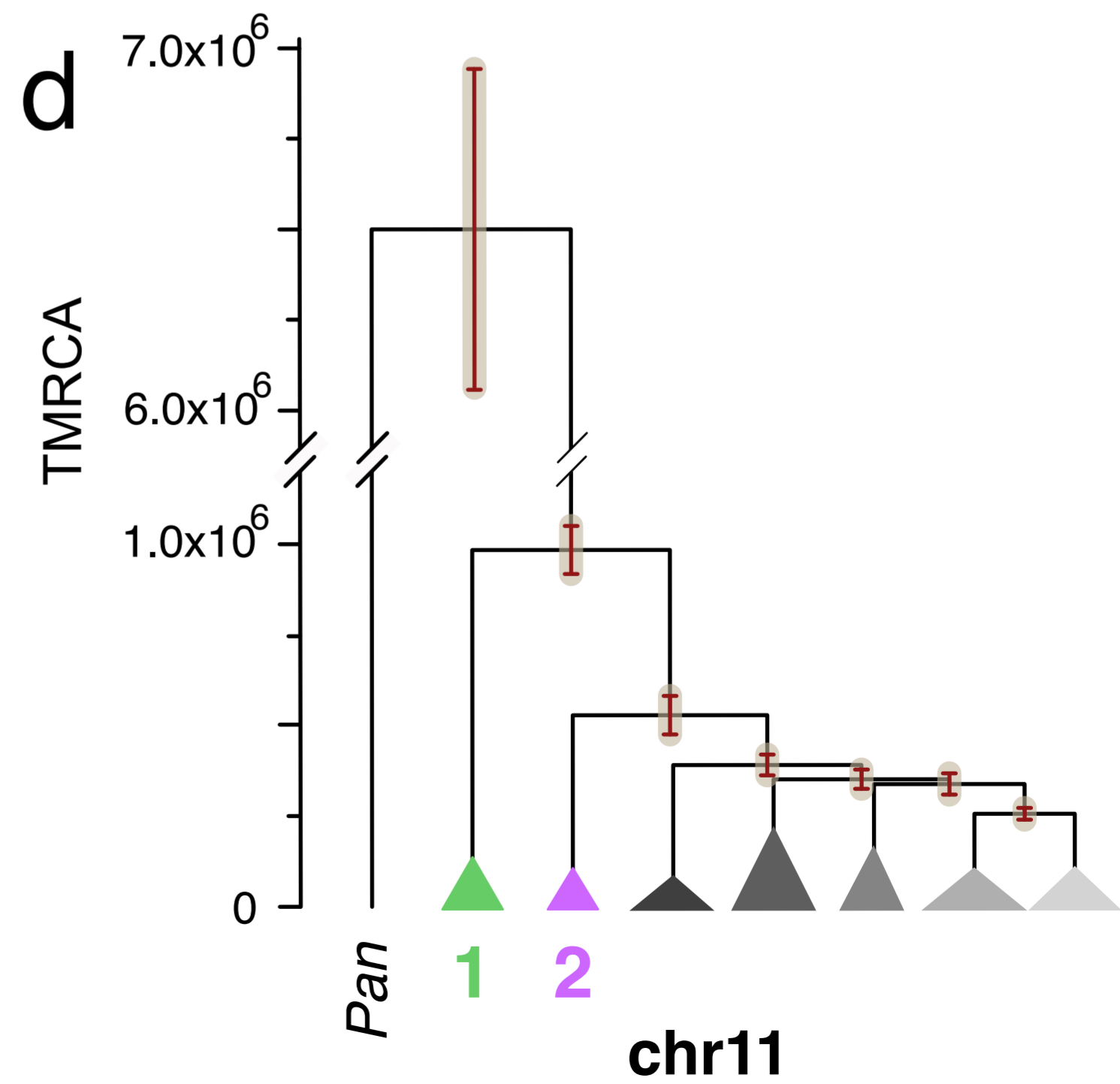

Supplement: Figure 3—source data 3. [file elife-42989-fig3-data3.pdf]

a

AFR  
AMR  
EAS  
EUR  
SAS

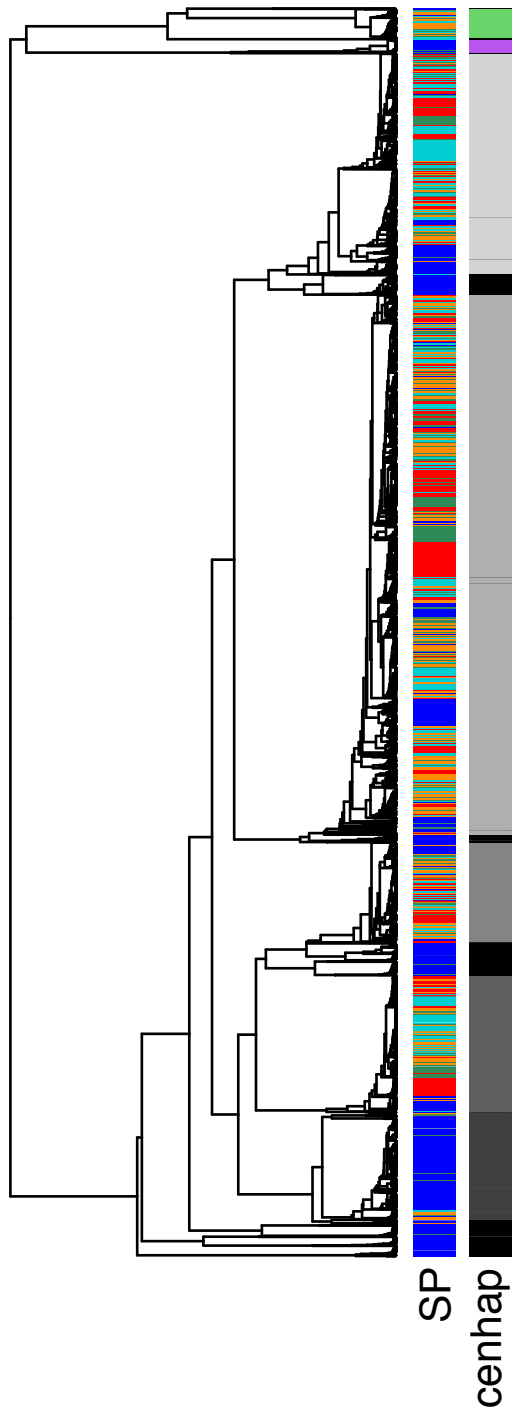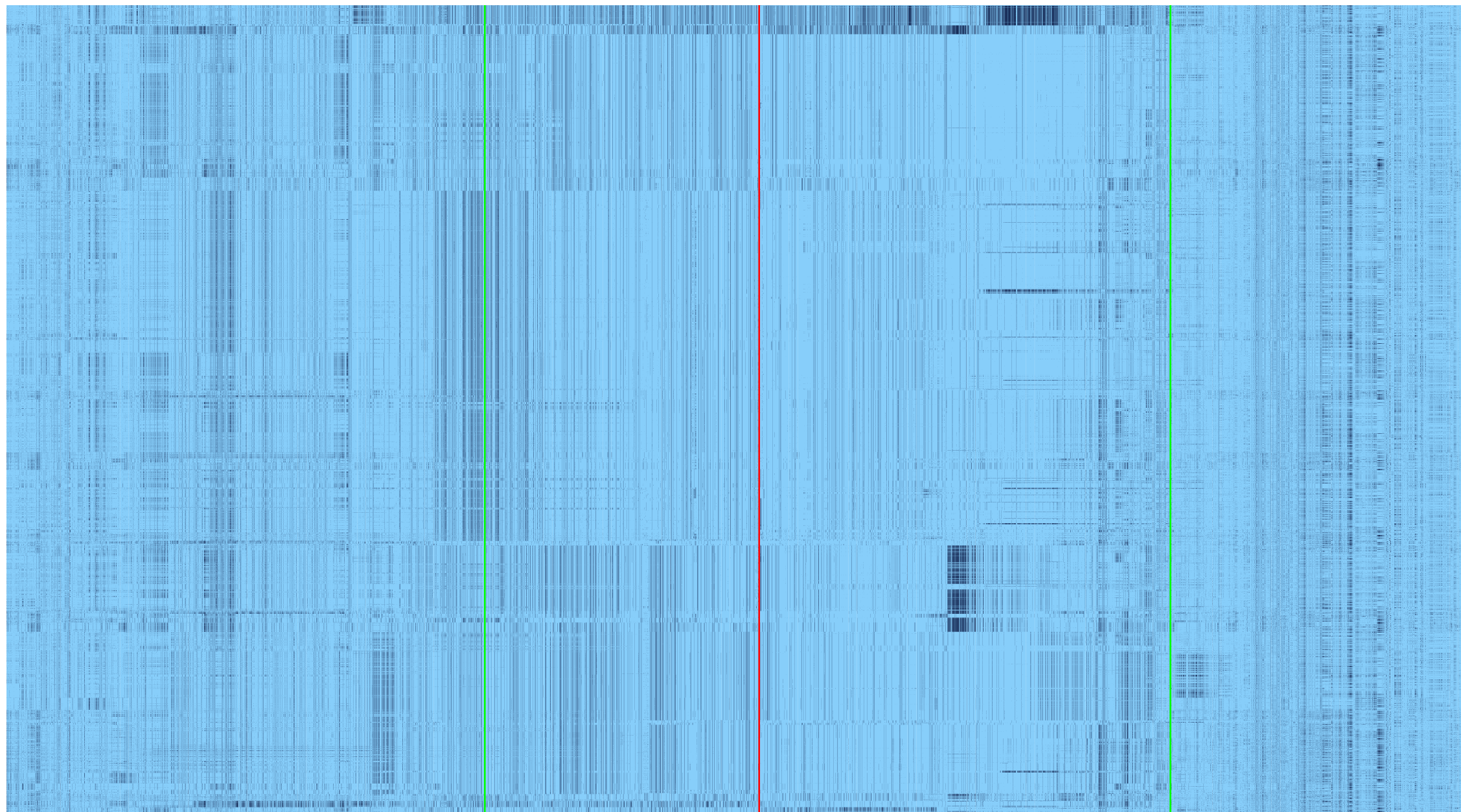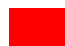

chr11

1  
2

Supplement: Figure 3—figure supplement 1—source data 1. [file elife-42989-fig3-figsupp1-data1.pdf]

a

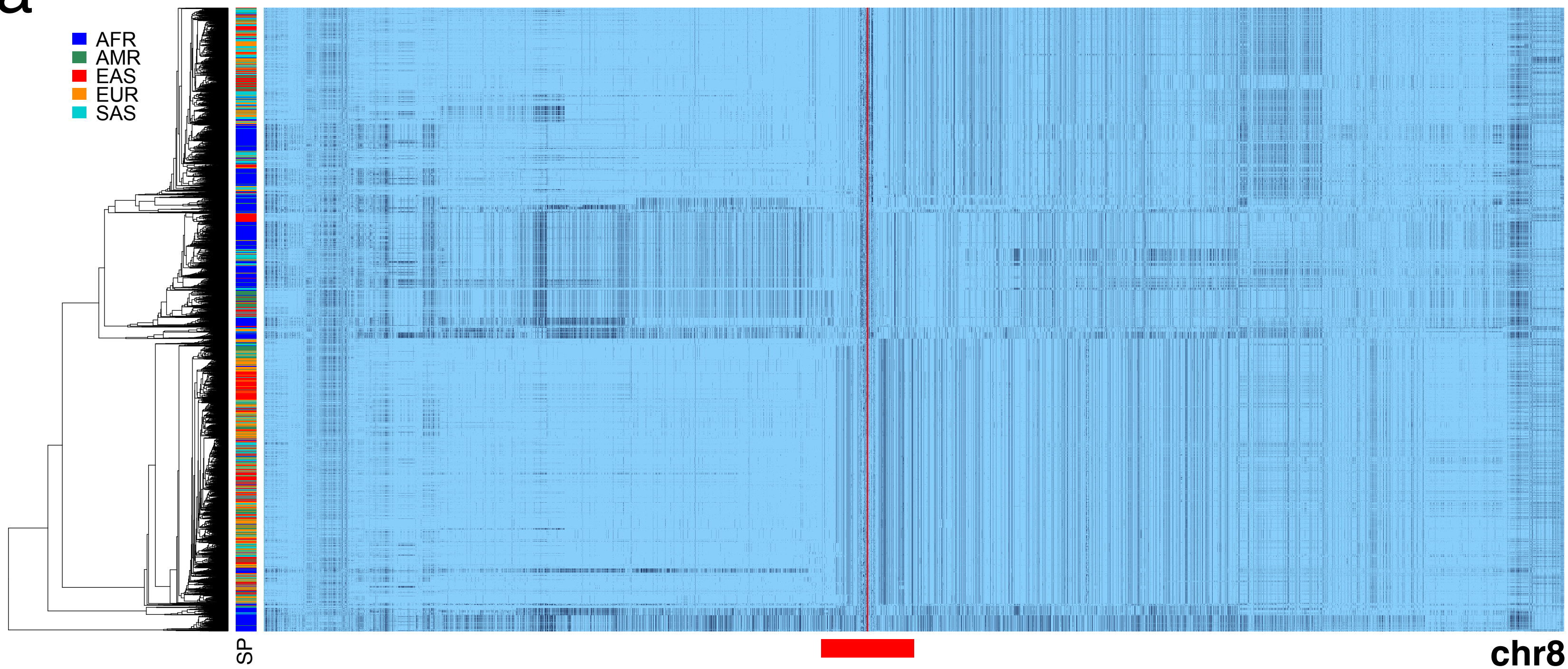

c

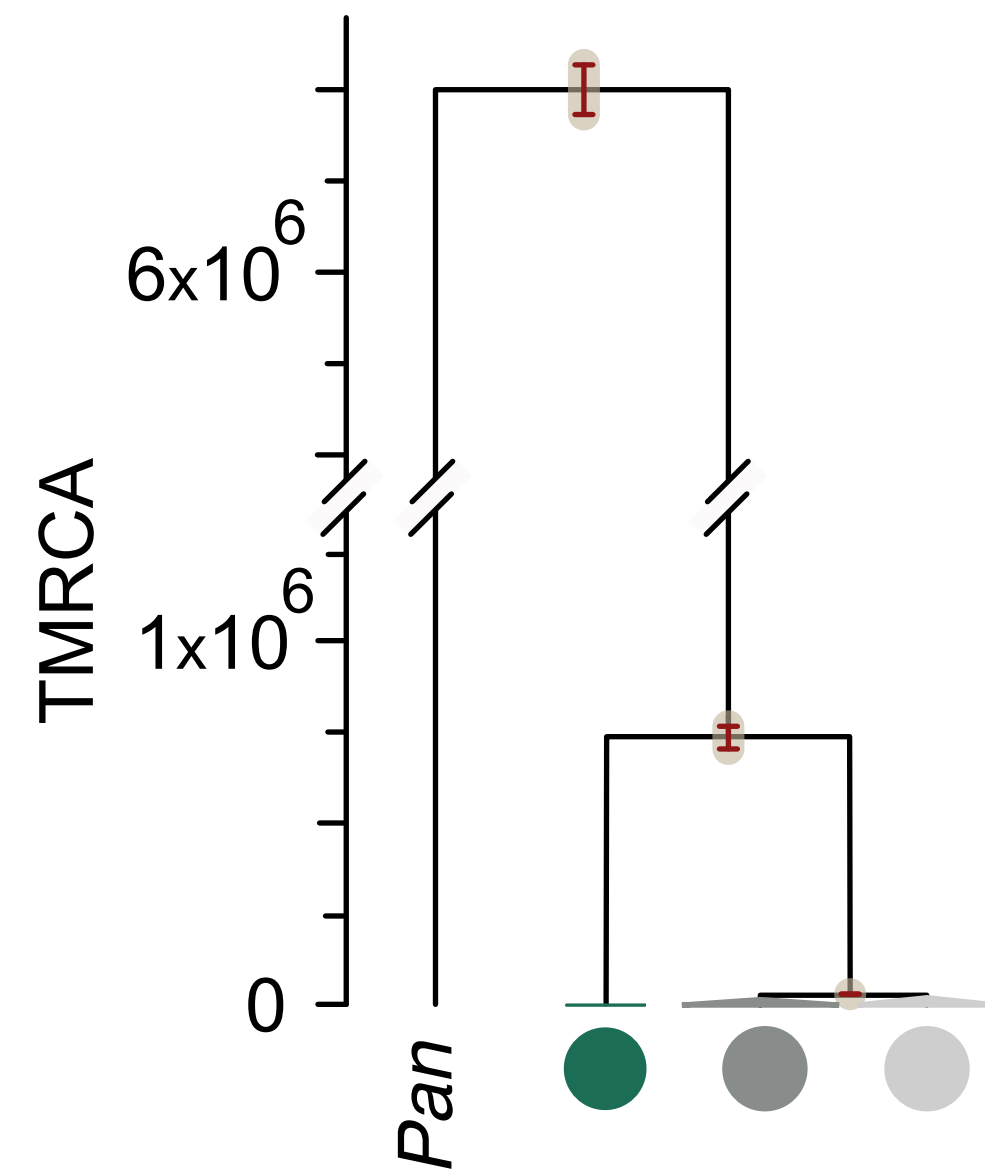

b

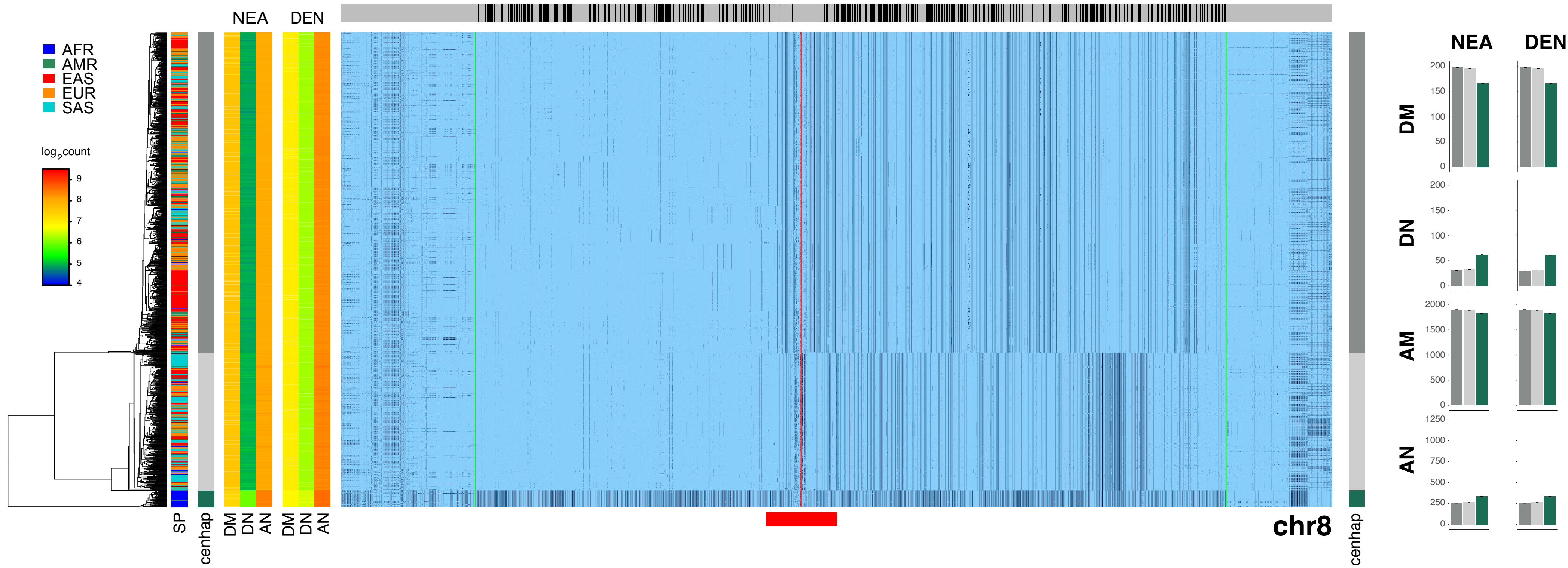

Supplement: Figure 3—figure supplement 2—source data 1. [file elife-42989-fig3-figsupp2-data1.pdf]

AFR  
AMR  
EAS  
EUR  
SAS

$\log_2$  count

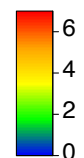

NEA

DEN

SP

DM  
DN  
AN

DM  
DN  
AN

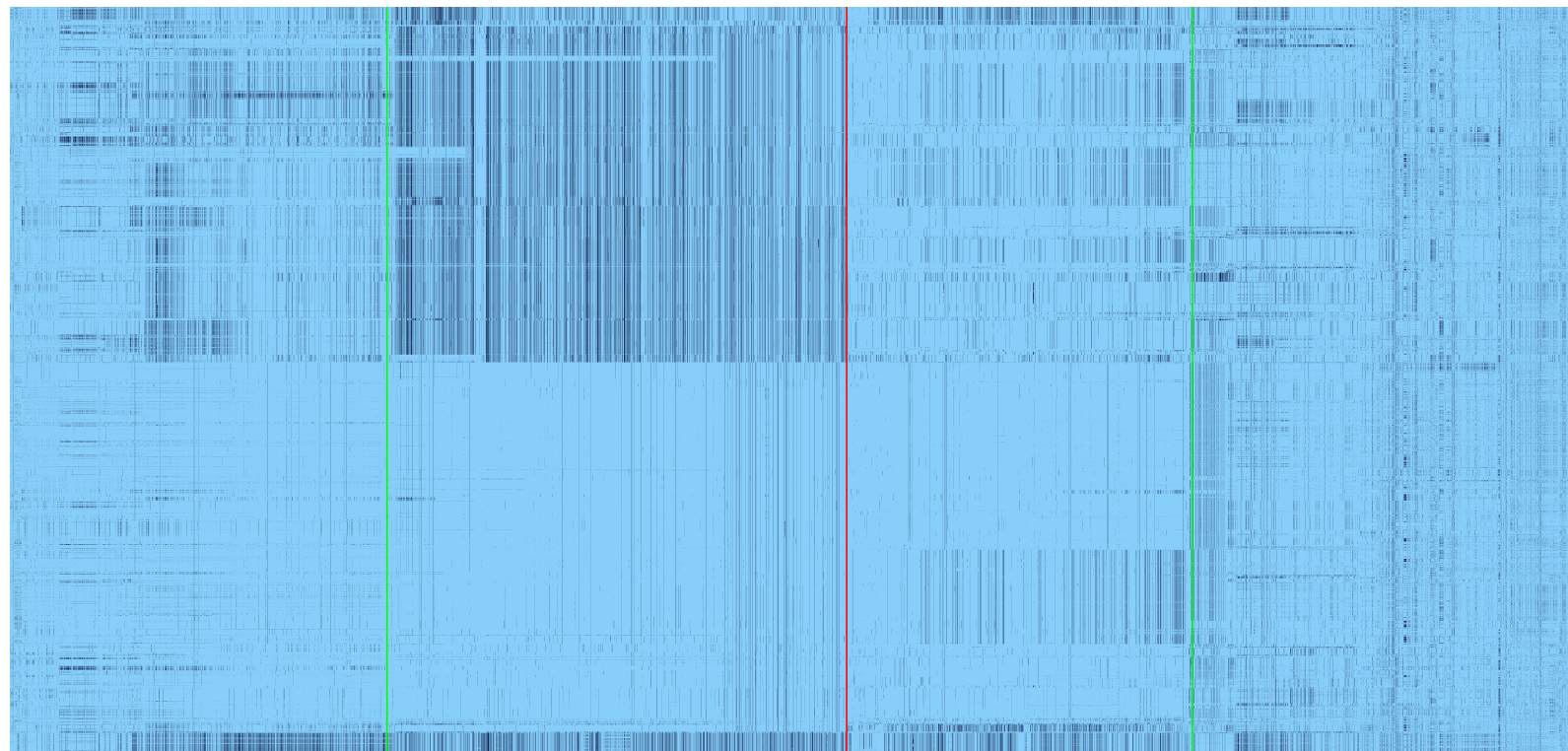

Supplement: Figure 3—figure supplement 3—source data 1. [file elife-42989-fig3-figsupp3-data1.pdf]

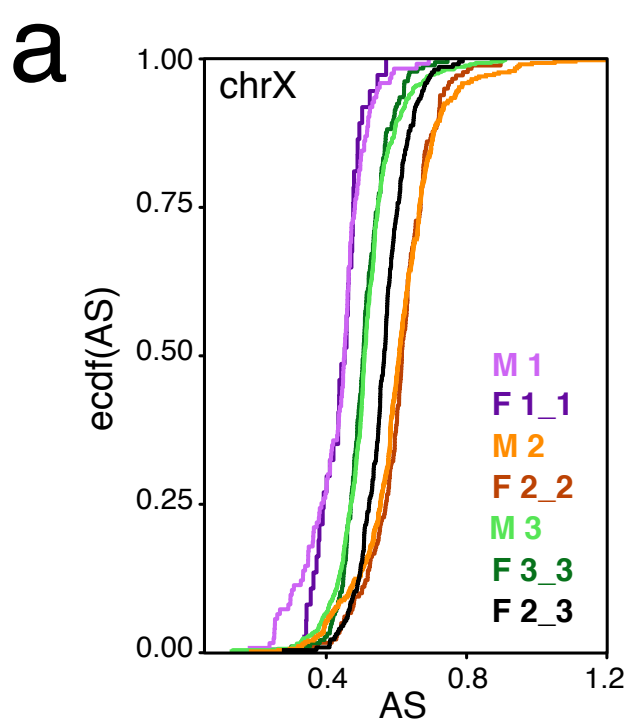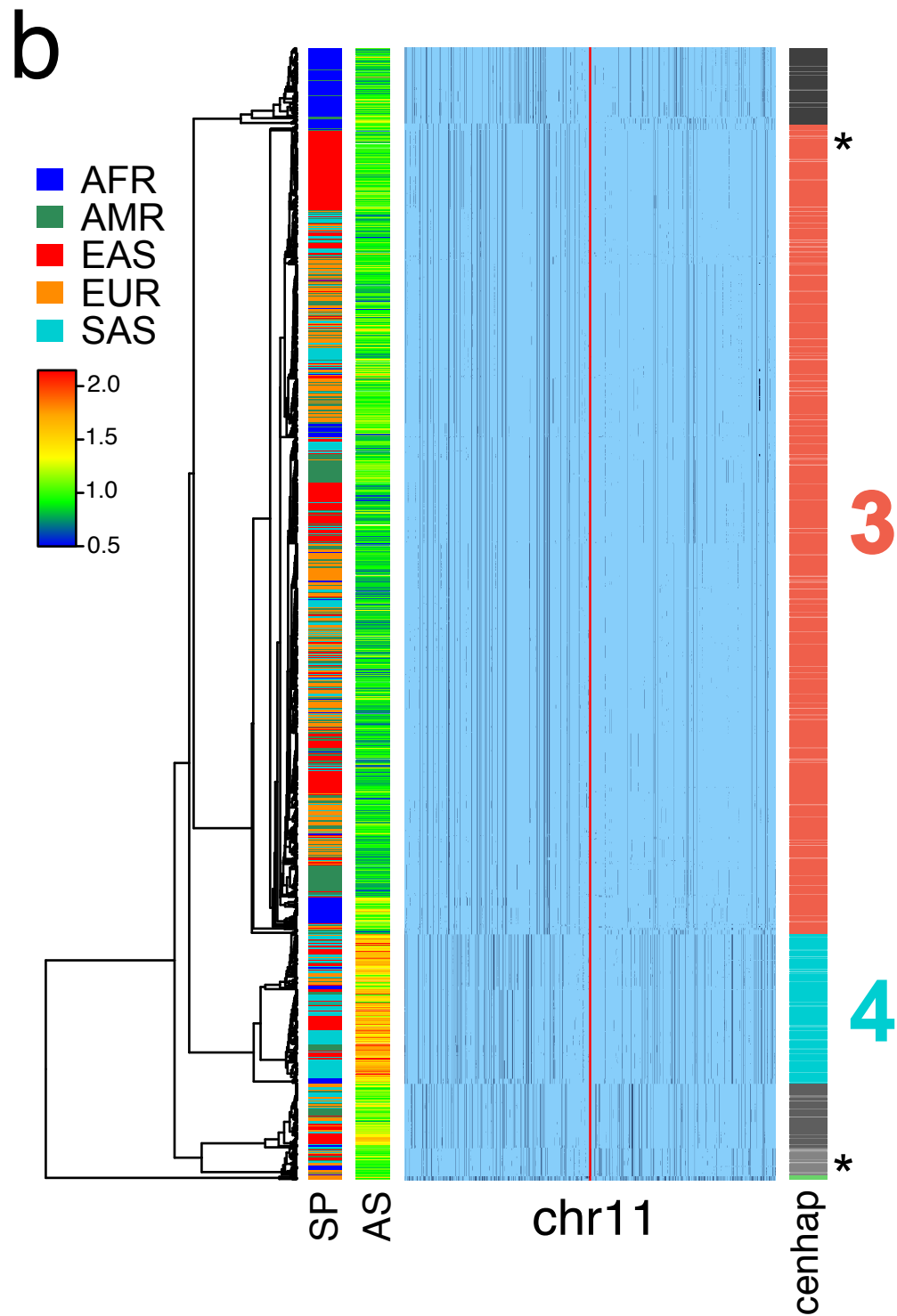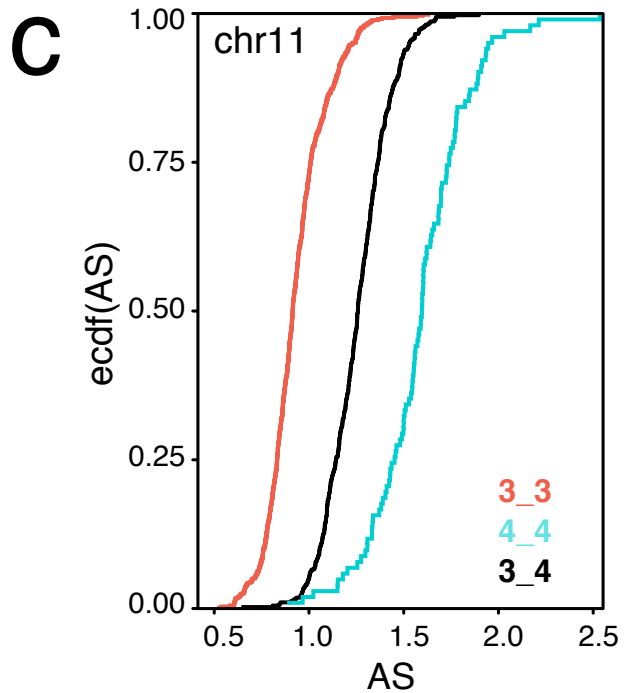

Supplement: Figure 4—source data 1. [file elife-42989-fig4-data1.pdf]

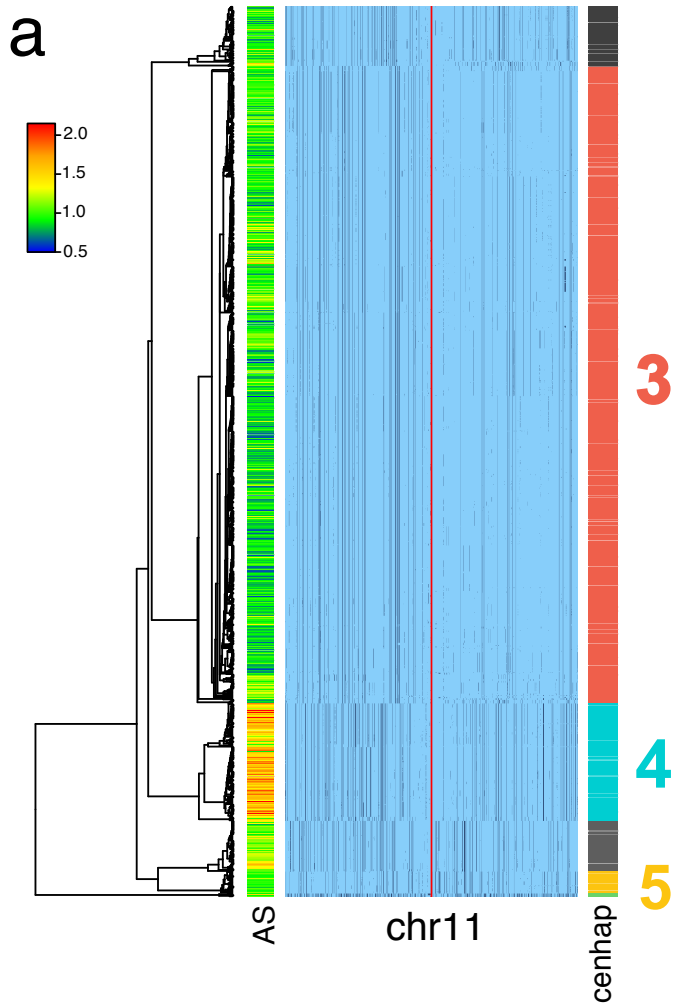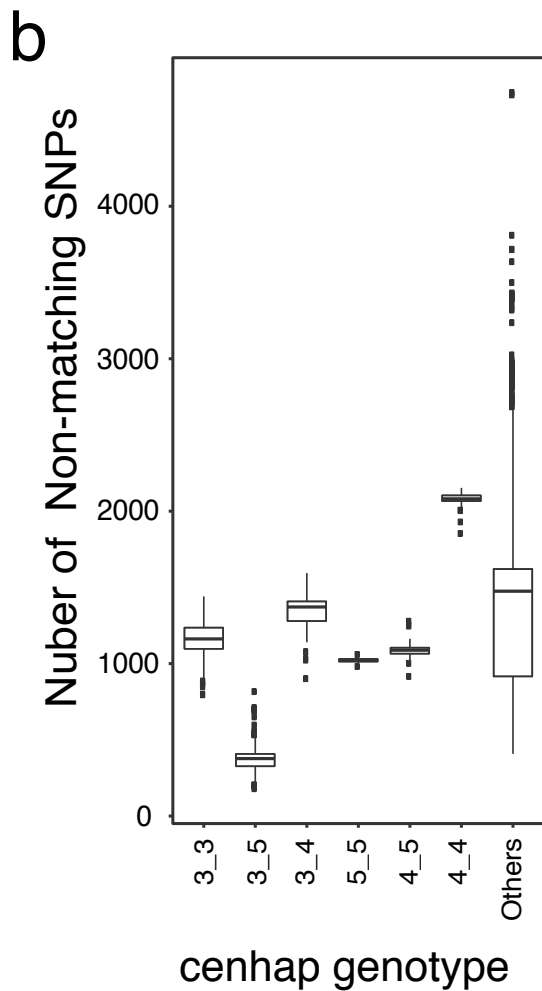

Supplement: Figure 4—figure supplement 1—source data 1. [file elife-42989-fig4-figsupp1-data1.pdf]
