# Supplementary material for: Haplotypes spanning centromeric regions reveal persistence of large blocks of archaic DNA
Source: eLife. 2019 Jun 25;8:e42989. doi: 10.7554/eLife.42989 (PMC6592686; doi:10.7554/eLife.42989)
Supplement: Figure 1—figure supplement 1—source data 1. [file elife-42989-fig1-figsupp1-data1.zip › full_resolution_Figure_1_figure_supplement_1.pdf]

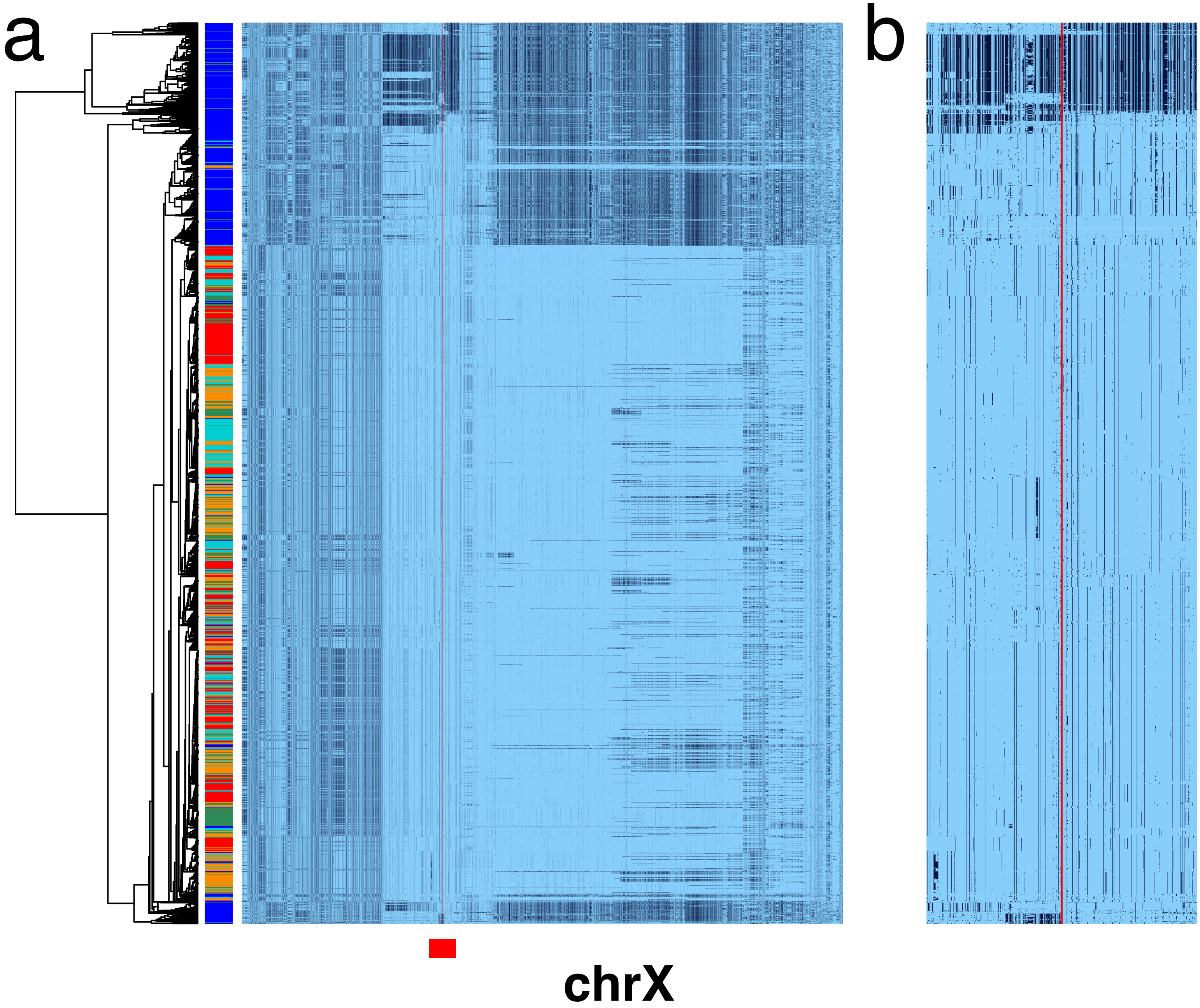
a
b
chrX
